# Supplementary material for: CircMEG3 inhibits telomerase activity by reducing Cbf5 in human liver cancer stem cells
Source: Mol Ther Nucleic Acids. 2020 Nov 17;23:310–23. doi: 10.1016/j.omtn.2020.11.009 (PMC7779543; doi:10.1016/j.omtn.2020.11.009)
Supplement: Document S1. Figures S1–S6 [file mmc1.pdf]

OMTN, Volume *23*

Supplemental Information

CircMEG3 inhibits telomerase activity by reducing Cbf5 in human liver cancer stem cells

Xiaoxue Jiang, Libo Xing, Yingjie Chen, Rushi Qin, Shuting Song, Yanan Lu, Sijie Xie, Liyan Wang, Hu Pu, Xin Gui, Tianming Li, Jie Xu, Jiao Li, Song Jia, and Dongdong Lu

Supplemental Figure

FigureS1

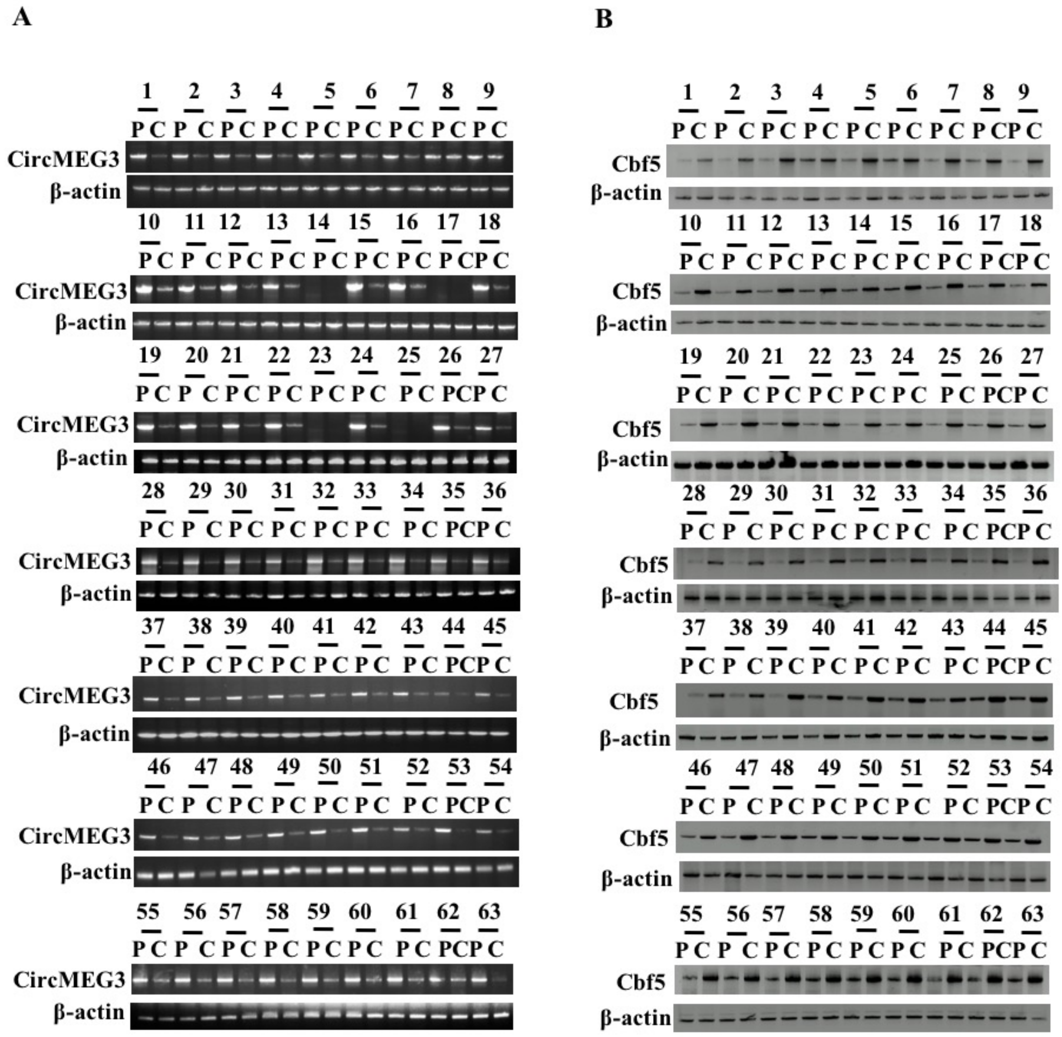

C

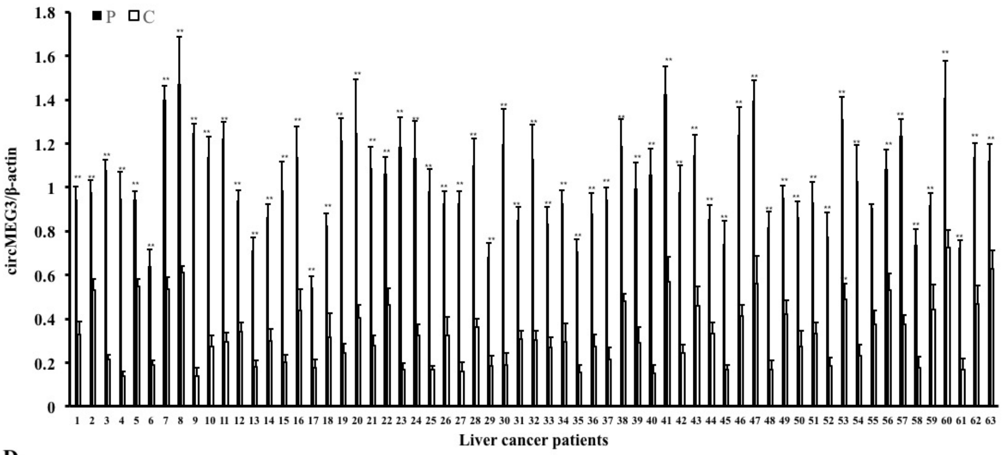

D

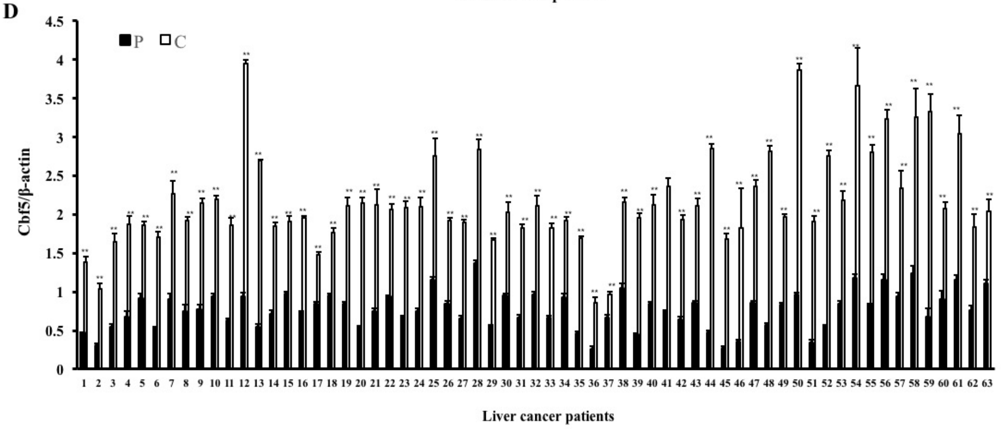

E

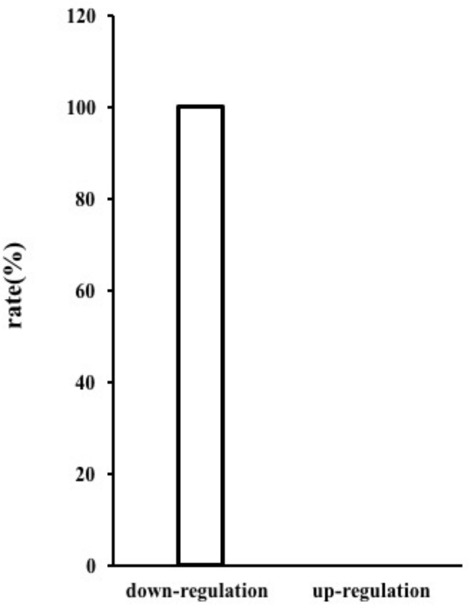

Cir c MEG3 expression in 63 cases of liver cancers

F

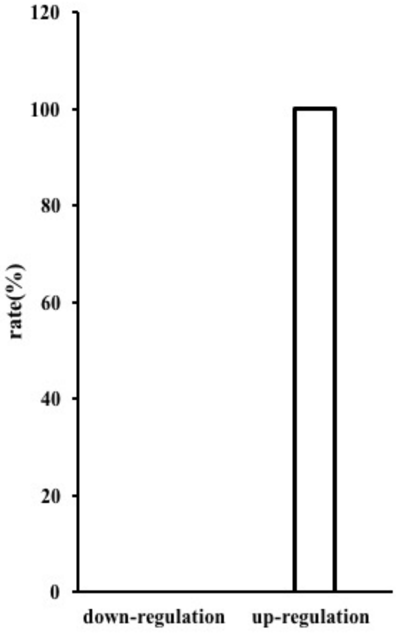

Cbff5 expression in 63 cases of liver cancers

FigureS1. Expression analysis of Circ MEG3 and telomerase-related gene Cbf5 in human liver cancer tissue. A. back-to-back RT-PCR analysis of CircMEG3 expression in liver cancer and its adjacent tissues. β-actin serves as an internal reference. B. Immunoblotting analysis of Cbf5 expression in liver cancer and its adjacent tissues. β-actin serves as an internal reference. C. Real-time back-to-back RT-PCR analysis of Circ MEG3 expression in liver cancer and its adjacent tissues. β-actin serves as an internal reference. D. Real-time RT-PCR analysis of Cbf5 mRNA in liver cancer and its adjacent tissues. β-actin serves as an internal reference.*C*: liver cancer tissues, *P* : adjacent noncancerous tissues. E. Circ MEG3 expression rate in liver cancer and its adjacent tissues. F. Cbf5 expression rate in liver cancer and its adjacent tissues.

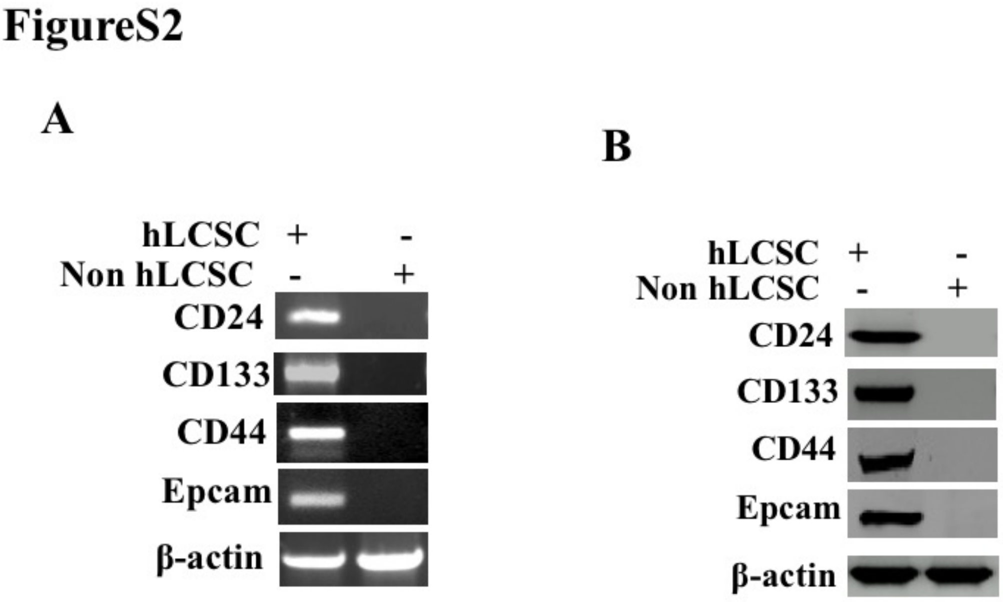

Figure S2 The identification of human liver cancer stem cells. RT-PCR (A) and Western blotting (B) analysis of CD133, CD44, CD24 and EpCAM. β-actin serves as an internal reference..

FigureS3

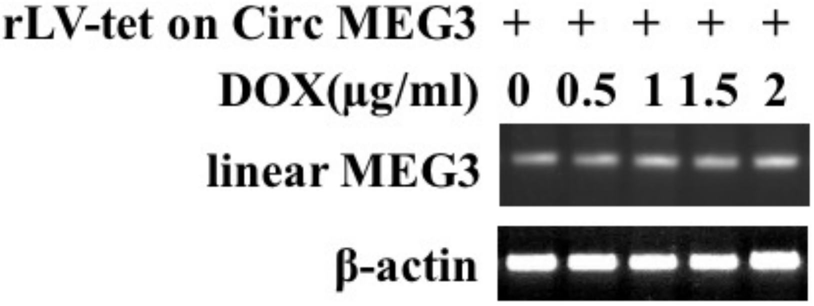

FigureS3 RT-PCR is used to detect linear MEG3 in human live cancer stem cell lines (hLCSCs) infected with rLV-tet on-CircMEG3 at different concentrations of DOX (0μg / ml, 0.5μg / ml, 1μg / ml, 1.5μg / ml, 2μg / ml) (E. semi-quantitative; F. quantitative). β-actin serves as an internal reference.

Figure S4

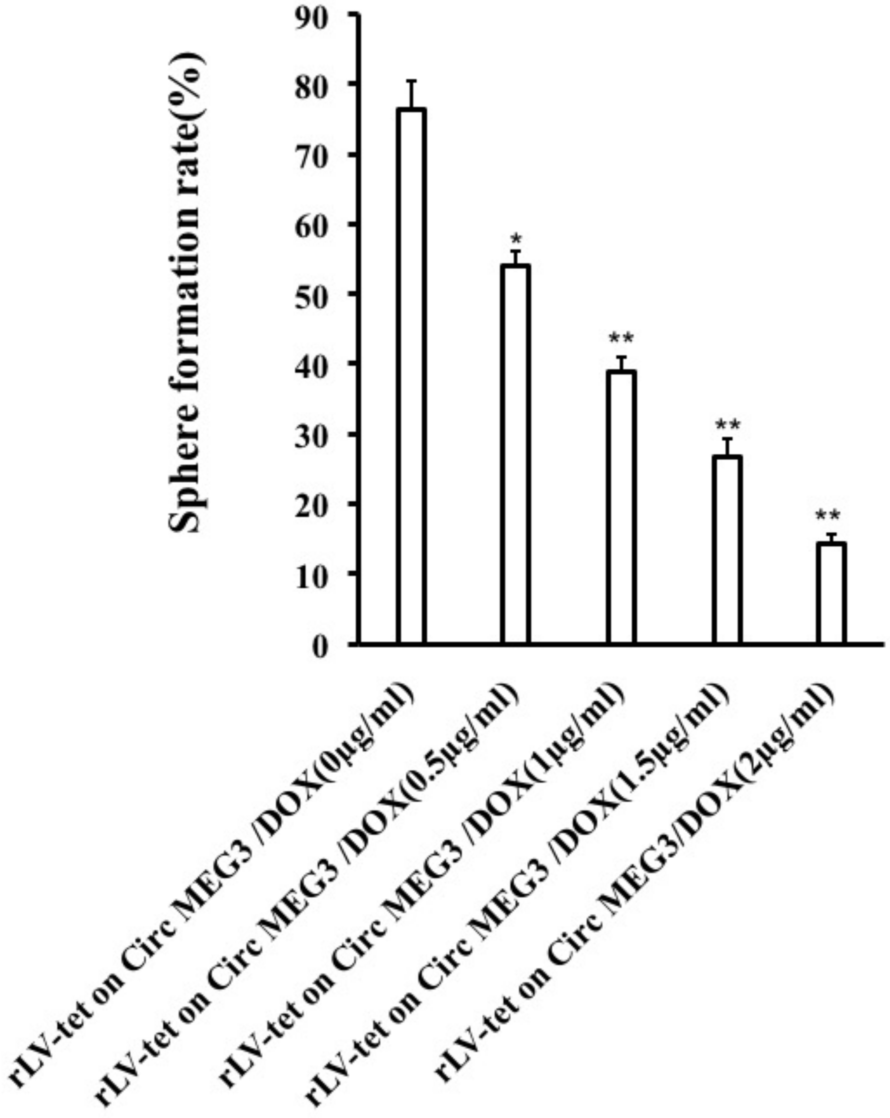

Figure S4 The analysis of human liver cancer stem cell sphere formation ability. The analysis of sphere formation rate. **, P <0.01 or *, P <0.05 means statistical difference is significant.

FigureS5

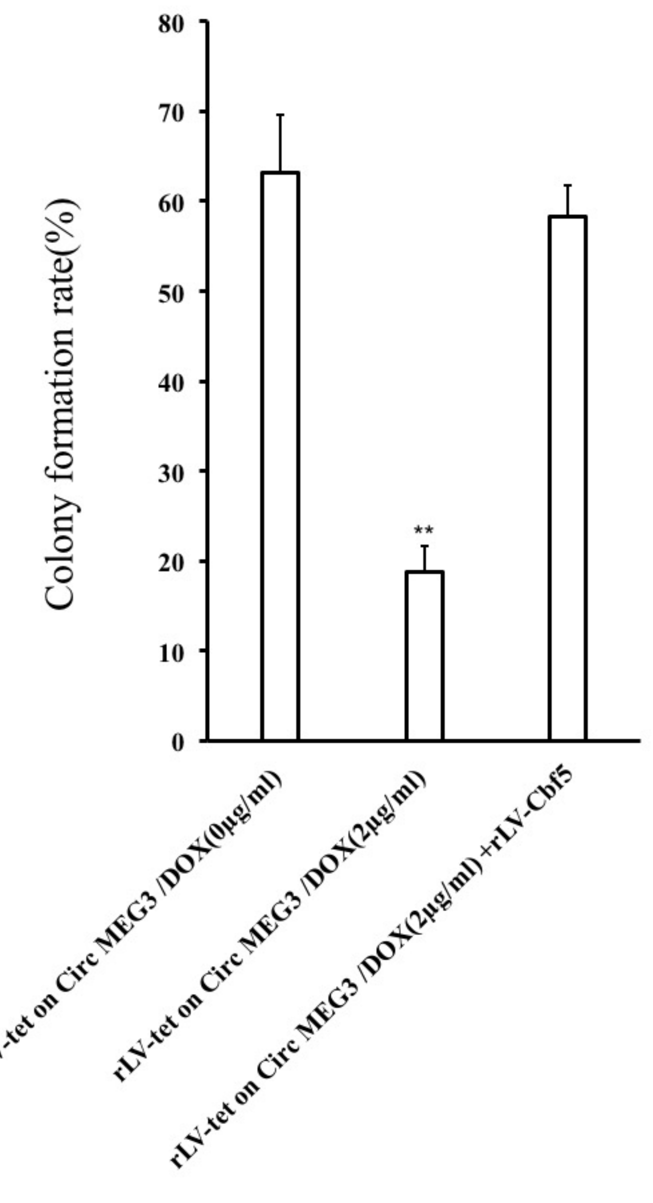

FigureS5. The analysis of of human liver cancer stem cell colony formation ability. **, P <0.01 or *, P <0.05 means statistical difference is significant.

FigureS6

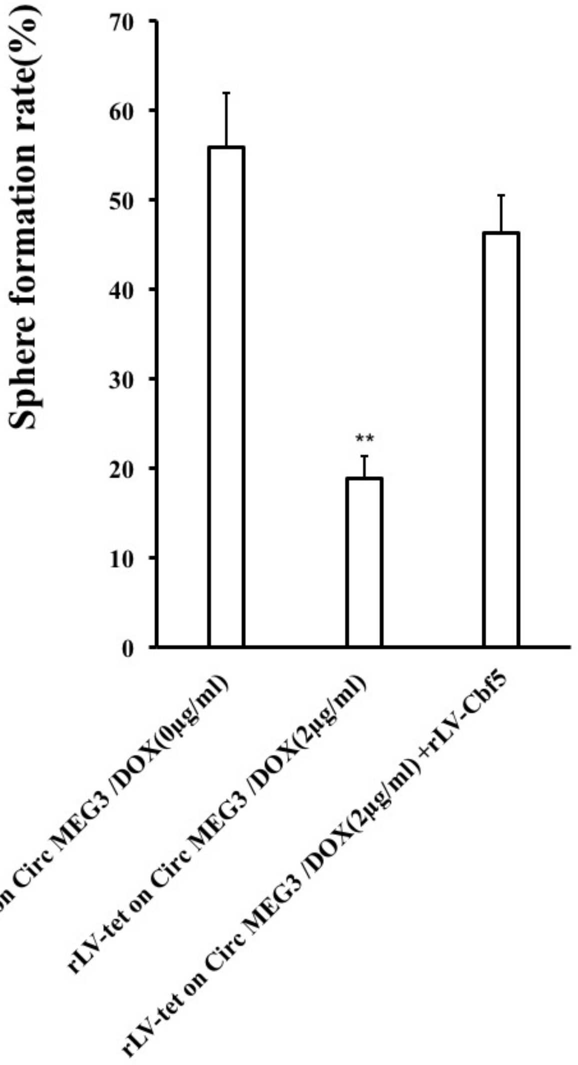

FigureS6 The analysis of human liver cancer stem cell sphere formation rate. **, P <0.01 or *, P <0.05 means statistical difference is significant.
