# Supplementary material for: CircMEG3 inhibits telomerase activity by reducing Cbf5 in human liver cancer stem cells
Source: Mol Ther Nucleic Acids. 2020 Nov 17;23:310–23. doi: 10.1016/j.omtn.2020.11.009 (PMC7779543; doi:10.1016/j.omtn.2020.11.009)
Supplement: Document S2. Article plus Supplemental Information [file mmc2.pdf]

Molecular Therapy
Nucleic Acids
Original Article

CircMEG3 inhibits telomerase activity by reducing Cbf5 in human liver cancer stem cells

Xiaoxue Jiang,[1,3] Libo Xing,[1,3] Yingjie Chen,[1,3] Rushi Qin,[1] Shuting Song,[1] Yanan Lu,[1] Sijie Xie,[1] Liyan Wang,[1] Hu Pu,[1] Xin Gui,[1] Tianming Li,[1] Jie Xu,[2] Jiao Li,[2] Song Jia,[2] and Dongdong Lu[1]

[1]Shanghai Putuo District People's Hospital, School of Life Science and Technology, Tongji University, Shanghai 200092, China; [2]School of Medicine, Tongji University, Shanghai 200092, China

Circular RNA (CircRNA) is a newly identified special class of non-coding RNA (ncRNA) that plays an important regulatory role in the progression of certain diseases. Herein, our results indicate that CircMEG3 is downregulated expression and negatively correlated with the expression of telomerase-related gene Cbf5 in human liver cancer. Moreover, CircMEG3 inhibits the growth of human liver cancer stem cells *in vivo* and *in vitro*. CircMEG3 inhibits the expression of m6A methyltransferase METTL3 dependent on HULC. Moreover, CircMEG3 inhibits the expression of Cbf5, a component of telomere synthetase H/ACA ribonucleoprotein (RNP; catalyst RNA pseudouracil modification) through METTL3 dependent on HULC. Thereby, CircMEG3 inhibits telomerase activity and shortens telomere lifespan dependent on HULC and Cbf5 in human liver cancer stem cell. Strikingly, increased Cbf5 abrogates the ability of CircMEG3 to inhibit malignant differentiation of human liver cancer stem cells. In summary, these observations provide important basic information for finding effective liver cancer therapeutic targets.

INTRODUCTION

It has been found that stem cells can be differentiated into malignant cells under unfavorable microenvironments.[1] Although most of the current support for malignant tumors stems from the malignant transformation of stem cells, the mechanism of stem cell deterioration is still controversial.[2,3] For example, METTL3-eIF3h promotes stem cell deterioration,[4] and farnesoid X receptor (FXR) regulates the proliferation of small-intestinal cancer stem cells (CSCs).[5–7] C-Myc is related to the malignant differentiation of leukemia stem cells.[8] Studies have found that JAK/STAT is highly activated in tumor stem cells.[9] Studies have confirmed that liver CSCs are closely related to the recurrence of liver cancer.[10] It is not clear what causes the accumulation of genetic errors of stem cells and changes in telomere function, which eventually evolve into malignant stem cells.

Circular RNA (CircRNA) is a newly identified special class of non-coding RNA (ncRNA) that plays an important regulatory role in the progression of certain diseases (such as tumors).[11,12] CircRNA can play the role of miRNA sponge.[13,14] Moreover, loop interactions between flanking introns can promote reverse splicing, thereby promoting the production of CircRNA.[15] For example, CircFOXP1 acts as a molecular switch that regulates Wnt and EGFR by acting as a sponge pad.[16] *hsa_circ_0072387* suppresses glycolysis of oral squamous cell carcinoma.[17] Circ0031288/hsa-miR-139-3p/Bcl-6 influences the invasion of cervical cancer HeLa cells.[18] Circ_001653 silencing promotes the cell proliferation.[19] CircRNACCDC66 regulates cisplatin resistance in gastric cancer via the miR-618/BCL2 axis.[20] Furthermore, Circ0000790 is involved in pulmonary vascular remodeling,[21] and CircRNF20 promotes breast cancer tumorigenesis and Warburg effect through miR-487a/HIF-1α/HK2.[22] Our previous research found that long ncRNA MEG3 can form circular MEG3 (CircMEG3), and it is lowly expressed in human liver cancer. MEG3 participates in the regulation of various growth,[23] for example, MEG3 silencing can induce mouse pluripotent stem cells.[23] MEG3 inhibits the activation of liver satellite cells,[24] and MEG3 as ceRNA regulates liver fat metabolism.[25] Studies have shown that the expression patterns of various transcriptional variants of MEG3 are tissue cell specific. For example, fetal liver cells express 12 MEG3 transcriptional variants.[26] Furthermore, the downregulated expression of MEG3 is related to gene hypermethylation.[27,28] MEG3 inhibits malignant proliferation of tumor cells dependent on P53.[29] In addition, MEG3 can also inhibit tumor growth in a P53-independent manner.[30–33]

It is well known that RNA m6A modification regulates RNA splicing, translocation, stability, and translation into protein. HULC is deregulated in cancer and acts as the potential biomarker and therapeutic target. Cbf5 is a component of telomere synthase H/ACA ribonucleoprotein (RNP). Our studies indicate that, first, CircMEG3 is downregulated expression and inversely correlated with the expression of telomerase-related gene Cbf5 in human liver cancer. Second, RNA

Received 30 July 2020; accepted 10 November 2020;
https://doi.org/10.1016/j.omtn.2020.11.009.

[3]These authors contributed equally

Correspondence: Dongdong Lu, Shanghai Putuo District People's Hospital, School of Life Science and Technology, Tongji University, Shanghai 200092, China.
E-mail: ludongdong@tongji.edu.cn

This is an open access article under the CC BY license (http://creativecommons.org/licenses/by/4.0/).

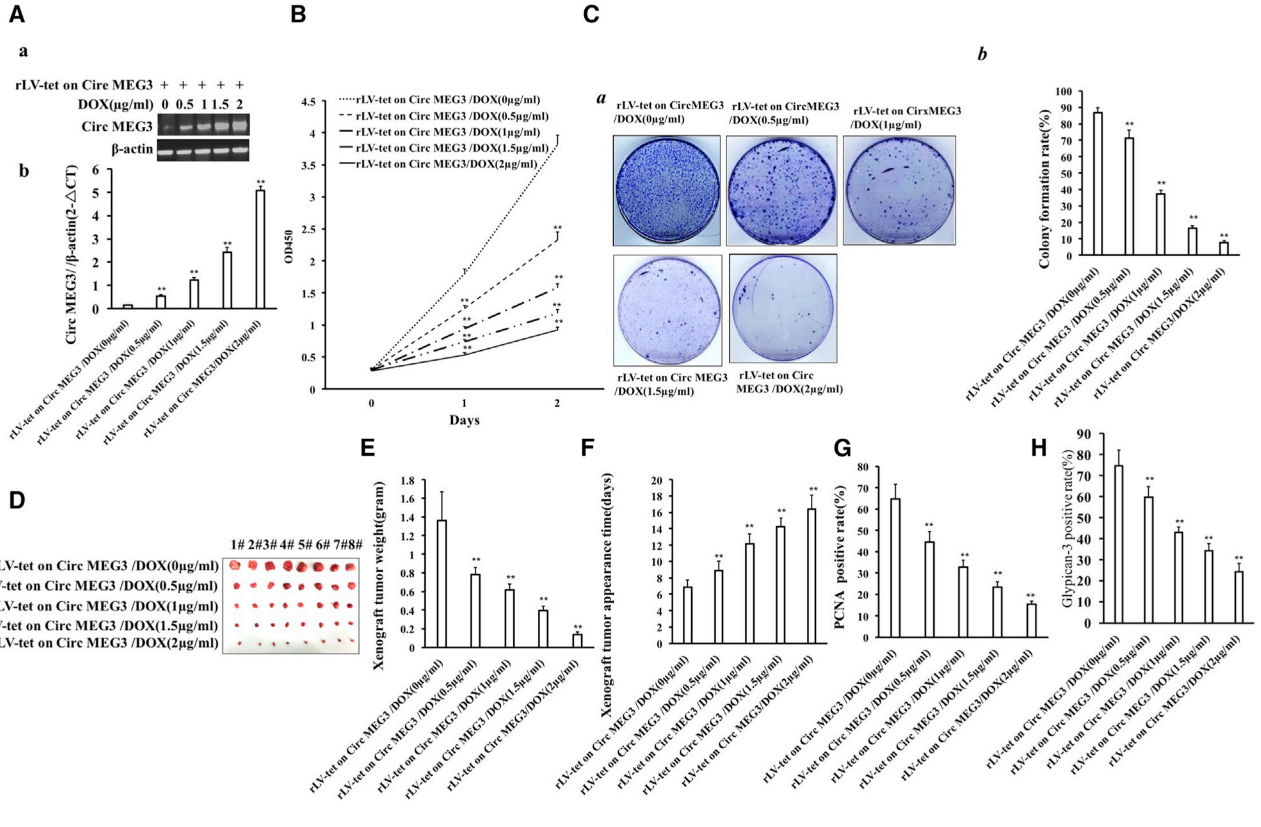

Figure 1. CircMEG3 inhibits the growth of human liver cancer stem cells *in vitro* and *in vivo*

(A) Back-to-back RT-PCR was used to detect CircMEG3 in the cells at different concentrations of DOX (0, 0.5, 1, 1.5, 2 μg/mL) (E: semiquantitative; F: quantitative). β-Actin serves as an internal reference. (B) Growth curve assay using CCK8. (C) The crystal violet staining method was used to determine the plate colony-forming ability. (Ca) Photograph of cell colonies. (Cb) The analysis of colony formation rate. (D) Photos of transplanted tumors (xenograft). (E) Comparison of the size (g) of transplanted tumors. (F) Comparison of the appearance time (days) of transplanted tumors. **p < 0.01 or *p < 0.05 means statistical difference is significant. (G) 4% formaldehyde-fixed, paraffin-embedded transplanted tumor tissue sections (4 μm) were subjected to anti-PCNA immunohistochemical staining. The comparison of PCNA-positive rates of transplanted tumors. (H) The comparison of Glypican-3-positive rates of transplanted tumors.

sequencing indicates that CircleMEG3 inhibits HULC, METTL3, and Cbf5 (data not shown). Moreover, Protein chip indicates HULC enhances METTL3 and Cbf5 (data not shown). In particular, HULC overexpression abrogates the actions of CircleMEG3 that inhibits METTL3 and Cbf5 (data not shown). Moreover, we have clearly demonstrated that MEG3 inhibits METTL3 through blocking HULC in human liver cancer (data not shown). In this study, we identify that CircMEG3 inhibits the expression of METTL3 dependent on HULC and therefore inhibits the expression of Cbf5 in human liver CSCs. Given that there are multiple functions of METTL3 in human cancers, we also have reasons to investigate whether CircMEG3 inhibits the expression of Cbf5 dependent on METTL3 by reducing the methylation modification of Cbf5 mRNA. Thereafter, we will consider whether CircMEG3 affects the telomere function dependent on Cbf5 in human liver CSCs.

In conclusion, we have explored the effect of CircMEG3 on malignant differentiation of human stem cells *in vivo* and *in vitro* and focused on the important role played by CircMEG3 in regulating telomere remodeling. These studies will play an important role in finding effective tumor therapeutic targets.

RESULTS

CircMEG3 expression is downregulated and negatively correlated with the expression of telomerase-related gene Cbf5 in human liver cancer

To investigate the relationship between the expression of CircMEG3 and telomerase-related gene Cbf5 in human liver cancer tissues, we analyzed samples from 63 human liver cancer patients. Back-to-back RT-PCR detection showed that CircMEG3 was downregulated in 63 human liver cancer specimens (Figures S1A, S1C, and S1E). Furthermore, immunoblotting analysis and RT-PCR showed that the expression of telomerase-related gene Cbf5 in liver cancer tissues was upregulated in 63 human liver cancer specimens (Figures S1B, S1D, and S1F). Collectively, these results suggest that CircMEG3 is downregulated expression and negatively correlated with the expression of telomerase-related gene Cbf5 in human liver cancer.

CircMEG3 inhibits the growth of human liver CSCs

To address the effect of CircMEG3 on the growth of human liver CSCs *in vivo* and *in vitro*, we isolated human liver CSCs from Huh7 cells using $CD133^+/CD44^+/CD24^+/EpCAM^+$ microbeads. In hLCSCs, CD133, CD44, CD24, and EpCAM were positively expressed, but not in non-hLCSCs (Figures S2A and S2B). In stable rLV-Tet-on-CircMEG3-hLCSCs cell lines of DOX groups (0, 0.5, 1, 1.5, and 2 μg/mL), CircMEG3 expression was significantly increased with increasing DOX concentration (Figures 1Aa and 1Ab). Moreover, there is no significant difference of linear MEG3 among the five groups (Figure S3). The cell growth ability was significantly decreased with increasing DOX concentration (24 h: p = 0.00623, 0.000053, 0.0077, 0.0017 < 0.01; 48 h: p = 0.000048, 0.0083, 0.00528, 0.00172 < 0.01 or < 0.05) (Figure 1B). The colony formation ability of LCSCs was significantly decreased with the increase of DOX concentration (86.92% ± 2.68% versus 71.07% ± 5.01%, p = 0.004123 < 0.01; 71.07% ± 5.01% versus 31.15% ± 2.21%, p = 0.007177 < 0.01; 31.15% ± 2.21% versus 16.45% ± 1.57%, p = 0.00254 < 0.01; 16.45% ± 1.57% versus 7.78% ± 1.03%, p = 0.00953 < 0.01) (Figures 1Ca and 1Cb). The sphere formation ability of LCSCs sphere was significantly decreased with the increase of DOX concentration (76.26% ± 4.09% versus 54.11% ± 2.08%, p = 0.01234 < 0.05; 54.11% ± 2.08% versus 38.92% ± 2.0%, p = 0.000199 < 0.01; 38.92% ± 2.0% versus 26.74% ± 2.69%, p = 0.00954 < 0.01; 26.74% ± 2.69% versus 14.2% ± 1.47%, p = 0.00999 < 0.01) (Figure S4). The average weight of transplanted tumors was significantly decreased with the increase of DOX concentration (1.36 ± 0.312 versus 0.7825 ± 0.075 g, p = 0.00134 < 0.01; 0.7825 ± 0.075 versus 0.6175 ± 0.062 g, p = 0.0011; 0.6175 ± 0.062 versus 0.396 ± 0.0466 g, p = 0.0000485 < 0.01; 0.396 ± 0.0466 versus 0.14125 ± 0.0269 g, p = 0.00000045 < 0.01) (Figures 1D and 1E). The average appearance time of transplanted tumors was significantly increased with increase of DOX concentration (6.875 ± 0.8345 versus 8.875 ± 1.126 days, p = 0.0026 < 0.01; 8.875 ± 1.126 versus 12.13 ± 1.25 days, p = 0.00077 < 0.01; 12.13 ± 1.25 versus 14.25 ± 1.04 days, p = 0.00077 < 0.00222; 14.25 ± 1.04 versus 16.375 ± 1.685 days, p = 0.00513 < 0.01) (Figure 1F). The positive rate of PCNA in transplanted tumors was significantly decreased with the increase of DOX concentration (64.61% ± 7.003% versus 44.603% ± 4.85%, p = 0.0000076 < 0.01; 44.603% ± 4.85% versus 32.67% ± 4.42%, p = 0.00027 < 0.01; 32.67% ± 4.42% versus 23.53% ± 2.52%, p = 0.00024 < 0.01; 23.53% ± 2.52% versus 15.56% ± 1.39%, p = 0.000064 < 0.01) (Figure 1G). Moreover, the expression of Glypican-3 (a differentiation marker of live cancer cell) in transplanted tumors was significantly decreased with the increase of DOX concentration (74.59% ± 7.46% versus 59.69% ± 4.97%, p = 0.0009767 < 0.01; 59.69% ± 4.97% versus 42.94% ± 2.55%, p = 0.0000063 < 0.01; 42.94% ± 2.55% versus 34.43% ± 3.37%, p = 0.000086 < 0.01; 34.43% ± 3.37% versus 24.23% ± 3.95%, p = 0.000888 < 0.01) (Figure 1H). Collectively, these results suggest that CircMEG3 inhibits the growth ability of human liver CSCs *in vitro* and *in vivo*.

CircMEG3 inhibits the expression of m6A methyltransferase METTL3

RNA m6A modification regulates RNA splicing, translocation, stability, and translation into protein. m6A is catalyzed by the RNA methyltransferase METTL3. HULC is deregulated in cancer and acts as the potential biomarker and therapeutic target. Moreover, we have demonstrated that linear MEG3 inhibits METTL3 through blocking HULC in human liver cancer (data not shown). To further explore the effect of CircMEG3 on the expression of m6A methyltransferase METTL3 via HULC in human liver CSCs, METTL3 expression was first detected in the rLV-Tet-on-CircMEG3-hLCSCs, and CircMEG3 was significantly increased with increasing DOX concentration in DOX groups (0, 0.5, 1, 1.5, and 2 μg/mL) (Figure 2A). The ability of RNA polymerase II to bind to the METTL3 promoter was significantly decreased with increasing DOX concentration (Figure 2B). The ability of RNA polymerase II to enter the METTL3 promoter-enhancer loop was significantly decreased with increasing DOX concentration (Figure 2C). However, excessive HULC abolished this function of CircMEG3 (Figure 2D). The binding capacity of RNA polymerase II to the METTL3 promoter probe was significantly decreased with increasing DOX concentration (Figure 2E); however, excessive HULC abolished this function of CircMEG3 (Figure 2F). The pEZX-MT-METTL3 promoter-Luc luciferase activity was significantly decreased with the increase of DOX concentration (317,434.79 ± 18,470.1 versus 166,769.97 ± 9,492.06, p = 0.00559 < 0.01; 166,769.97 ± 9,492.06 versus 91,794.27 ± 6,072.62, p = 0.00078 < 0.01; 91,794.27 ± 6,072.62 versus 37,402.86 ± 5,199.99, p = 0.00649 < 0.01; 37,402.86 ± 5,199.99 versus 11,032.89 ± 1,239.77, p = 0.00529 < 0.01) (Figure 2G); however, excessive HULC abrogated this function of CircMEG3 (57,9365.78 ± 30,045.72 versus 32,670.41 ± 7,101.41, p = 0.000305 < 0.01; 579,365.78 ± 30,045.72 versus 514,115.08 ± 50,226.09, p = 0.1422 > 0.05) (Figure 2H). The expression of METTL3 was significantly decreased with increasing DOX concentration (Figures 2I and 2J). However, excessive HULC abolished this function of CircMEG3 (Figures 2K and 2L). Collectively, these results suggest that CircMEG3 inhibits the expression of METTL3 dependent on HULC in human liver CSCs.

CircMEG3 inhibits the expression of Cbf5, a component of telomere synthase H/ACA RNP

Given the multiple functions of METTL3 in human cancers, we considered investigating whether CircMEG3 inhibits the expression of Cbf5, a component of human liver CSC telomere synthase H/ACA RNP (catalytic RNA pseudourea modification); dependent on METTL3, the methylation modification of Cbf5 mRNA was detected in the DOX groups (0, 0.5, 1, 1.5, and 2 μg/mL) of rLV-Tet-on-CircMEG3-hLCSCs. The binding capacity of METTL3 and Cbf5 mRNA (a component of the telomere synthase H/ACA RNP [catalytic RNA pseudouracil modification]) was significantly decreased with increasing DOX concentration (Figure 3A). The binding capacity of METTL3 to Cbf5 mRNA probe was significantly decreased with increasing DOX concentration (Figure 3B). The level of methylation modification of Cbf5 mRNA was significantly decreased with increasing DOX concentration (Figure 3C); however, excessive HULC abolished this function of CircMEG3 (Figure 3D). The level of methylation modification of Cbf5 mRNA was significantly decreased in the rLV-Tet-on-CircMEG3/DOX (2 μg/mL) group

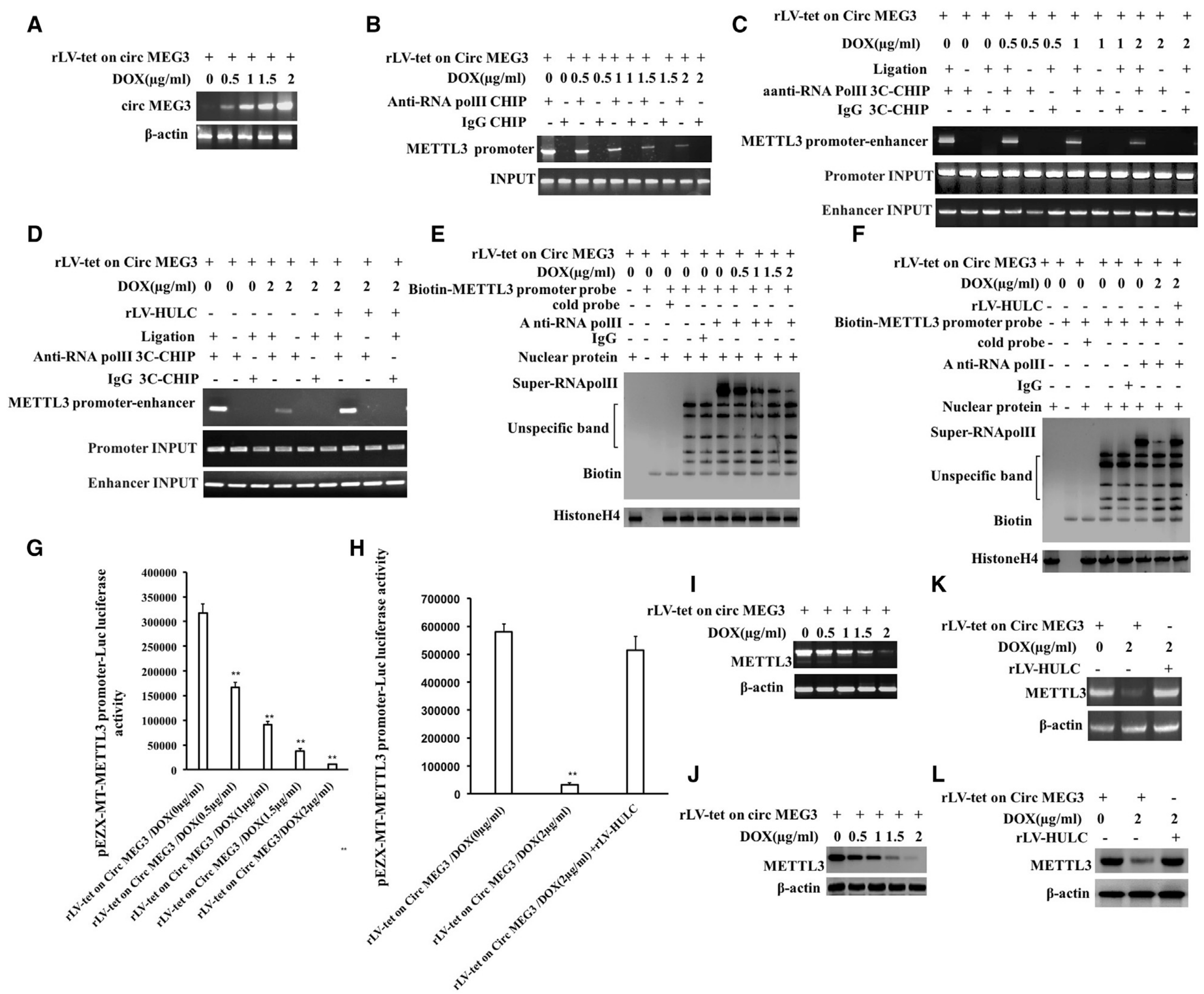

Figure 2. CircMEG3 inhibits the expression of m6A methyltransferase METTL3

(A) Back-to-back reverse transcription polymerase chain reaction (back-to-back RT-PCR) analysis was performed with CircMEG3 primers. β-Actin was used as an internal reference gene. (B) The chromatin immunoprecipitation (ChIP) was performed using anti-RNA polymerase II (anti-RNA Pol II). IgG ChIP was used as a negative control, and the METTL3 promoter primers were used as an internal reference (INPUT). (C and D) The cells were cross-linked with formaldehyde and then captured by chromosome architecture (3C)-ChIP using anti-RNA Pol II. IgG ChIP-3C was used as a negative control, and the products amplified by independent primers designed by METTL3 promoter and enhancer were used as internal reference (INPUT). (E and F) Super-DNA-protein complex gel migration assay using biotin-labeled METTL3 *cis*-element probe and anti-RNA Pol II, anti-Biotin. IgG super-EMSA was a negative control. (G and H) The pEZX-MT-METTL3-Luc luciferase reporter activity was measured. $^{**}p < 0.01$, $^{*}p < 0.05$. (I and K) The RT-PCR analysis was performed using METTL3 primers. β-Actin was used as an internal reference gene. (J and L) The total protein was subjected to western blotting using anti-METTL3. β-Actin was an internal reference gene.

compared with the rLV-Tet-on-CircMEG3/DOX (0 μg/mL) group; however, excessive METTL3 abrogated this function of CircMEG3 (Figure 3E). pEZX-MT-Cbf5 3′ UTR-Luc luciferase activity was significantly decreased with increasing DOX concentration (630,853.94 ± 57,010.88 versus 285,668.56 ± 27,879.33, p = 0.00123 < 0.01; 285,668.56 ± 27,879.33 versus 115,340.53 ± 7,176.01, p = 0.00262 < 0.01; 115,340.53 ± 7,176.01 versus 67,179.89 ± 1,626.63, p = 0.00524 < 0.01; 67,179.89 ± 1,626.63 versus 23,566.23 ± 2,167.09, p = 0.00524 < 0.01) (Figure 3F); however, excessive HULC abolished this function of CircMEG3 (414,507.92 ± 37,013.01 versus 88,071.49 ± 11,633.27, p = 0.00141 < 0.01; 414,507.92 ± 37,013.01 versus 375,032.89 ± 33,702.8, p = 0.09515 > 0.05) (Figure 3G). The transcriptional and translational capabilities of Cbf5 were significantly decreased with increasing DOX concentration (Figures 3H and 3I); however, excessive HULC abrogated this function of CircMEG3 (Figures 3J and 3K). Collectively, these results suggest that CircMEG3 inhibits the expression of Cbf5 through METTL3 dependent on HULC in human liver CSCs.

CircMEG3 inhibits telomerase activity and shortens telomere life in human liver CSCs

Given that CircMEG3 inhibits the expression of Cbf5, we will consider whether CircMEG3 affects the telomere function dependent on Cbf5 in human liver CSCs. In the DOX groups (0, 0.5, 1, 1.5, and 2 μg/mL) of rLV-Tet-on-CircMEG3-hLCSCs, the binding capacity of Cbf5 to H/ACA was significantly decreased with increasing DOX concentration (Figure 4A). The binding ability of Cbf5 to H/ACA probe was significantly decreased with increasing DOX concentration (Figure 4B). The binding capacity of TERT to TERC was significantly decreased with increasing DOX concentration (Figure 4C). The binding ability of TERT to TERC probes was significantly decreased with increasing DOX concentration (Figure 4D). The binding ability of TERT to the telomerase complex component proteins Cbf5, TCAB1, Reptin, and Pontin was significantly decreased with increasing DOX concentration (Figure 4E). The binding ability of TERT, Cbf5, TCAB1, Reptin, and Pontin to the telomerase RNA (TERC) H/ACA probe was significantly decreased with increasing DOX concentration (Figure 4F). Quantitative telomerase activity assay (TRAP) detection showed that telomerase activity of liver CSCs was significantly decreased with increasing DOX concentration (0.087 ± 0.0074 versus 0.0411 ± 0.0051, $p = 0.00218 < 0.01$; 0.0411 ± 0.0051 versus 0.0122 ± 0.0013, $p = 0.00355 < 0.01$; 0.0122 ± 0.0013 versus 0.0052 ± 0.0051, $p = 0.00739 < 0.01$; 0.0052 ± 0.0051 versus 0.0014 ± 0.000173, $p = 0.00195 < 0.01$) (Figure 4Ga). However, excessive HULC abolished this function of CircMEG3 (0.078 ± 0.006245 versus 0.0026 ± 0.000458, $p = 0.001 < 0.01$; 0.078 ± 0.006245 versus 0.0683 ± 0.01569, $p = 0.2167 > 0.05$) (Figure 4Gb). PCR amplification-Southern blot and quantitative PCR (qPCR) results showed that the length of telomere of liver CSCs was significantly decreased with the increase of DOX concentration (5.71 ± 0.68 versus 3.11 ± 0.135, $p = 0.0073 < 0.01$; 3.11 ± 0.135 versus 2.04 ± 0.07, $p = 0.00375 < 0.01$; 2.04 ± 0.07 versus 1.12 ± 0.11, $p = 0.00566 < 0.01$; 1.12 ± 0.11 versus 0.71 ± 0.032, $p = 0.0091 < 0.01$) (Figures 4Ha and 4Hb); however, excessive HULC abolishes this function of CircMEG3 (5.63 ± 0.89 versus 0.993 ± 0.121, $p = 0.00695 < 0.01$; 5.63 ± 0.89 versus 5.006 ± 0.61339, $p = 0.27265 > 0.05$) (Figures 4Ia and 4Ib). Collectively, these results suggest that CircMEG3 inhibits telomerase activity and shortens telomere life dependent on HULC and Cbf5 in human liver CSCs.

Increased Cbf5-telomerase activity abrogates the ability of CircMEG3 to inhibit malignant differentiation of human liver CSCs

To confirm whether CircMEG3 inhibits the ability of malignant differentiation of human liver CSCs by inhibiting Cbf5-telomerase activity, we conducted a rescue test. Compared with the DOX (0 μg/mL) group, the expression of CircMEG3 was increased, and the expression of Cbf5 was decreased in the DOX (2 μg/mL) treatment group, and both expressions of CircMEG3 and Cbf5 were increased in the DOX (2 μg/mL) + rLV-Cbf5 group (Figures 5A and 5B). In the DOX groups (0, 0.5, 1, 1.5, and 2 μg/mL) of rLVX-Tet-on-CircMEG3-hLCSCs, the DNA damage repair ability was significantly increased with increasing DOX concentration (Figure 5Ca). However, compared with the DOX (0 μg/mL) group, the DNA damage repair ability was not significantly altered in the DOX (2 μg/mL) + rLV-Cbf5 treatment group (Figure 5Cb). Using alisertib to induce cellular DNA damage, we detected the level of the DNA damage marker rH2AX (S139) by immunoblotting, and the results showed that H2AX (S139) expression was significantly reduced in the DOX (2 μg/mL) group compared with the DOX (0 μg/mL) group; however, it was significantly altered in the DOX (2 μg/mL) + rLV-Cbf5 group compared with the DOX (0 μg/mL) group (Figure 5Da). The immunostaining with anti-H2AX (S139) results showed that the DNA damage repair ability was significantly increased in the DOX (2 μg/mL) group compared with the DOX (0 μg/mL) group (40.66 ± 4.36 versus 17.08 ± 2.22, $p = 0.00452 < 0.01$); however, it was not significantly altered in the DOX (2 μg/mL) + rLV-Cbf5 group compared with the DOX (0 μg/mL) group (40.66 ± 4.36 versus 44.63 ± 9.81, $p = 0.1682$, $p > 0.05$) (Figures 5Db and 5Dc). Microsatellite instability (MSI) analysis showed that MSI was significantly decreased in the DOX (2 μg/mL) group compared with the DOX (0 μg/mL) group; however, it was not significantly altered in the DOX 2 μg/mL + rLV-Cbf5 group compared with the DOX (0 μg/mL) group (Figure 5E). The expression of the chromatin reprogramming factors Oct4, Sox2, KLF4, and Nanog was significantly reduced in the DOX (2 μg/mL) group compared with the DOX (0 μg/mL) group; however, it was not significantly altered in the DOX (2 μg/mL) + rLV-Cbf5 group compared with the DOX (0 μg/mL) group (Figure 5F). The expression of the chromatin instability factors KIF2B and KIF2C was significantly reduced in the DOX (2 μg/mL) group compared with the DOX (0 μg/mL) group; however, it was significantly not altered in the DOX (2 μg/mL) + rLV-Cbf5 group compared with the DOX (0 μg/mL) group (Figure 5G). The expression of oncogenes C-myc, CDK4, and H-Ras in the DOX (2 μg/mL) group compared with the DOX (0 μg/mL) group; however, it was significantly not altered in the DOX (2 μg/mL) + rLV-Cbf5 group compared with the DOX (0 μg/mL) group (Figure 5H). The cell proliferation ability was significantly reduced in the DOX (2 μg/mL) group compared with the DOX (0 μg/mL) group (24 h: $p = 0.00497 < 0.01$; 48 h: $p = 0.00893 < 0.01$). However, it was significantly not altered in the DOX (2 μg/mL) + rLV-Cbf5 group compared with the DOX (0 μg/mL) group (24 h: $p = 0.2977 > 0.05$; 48 h: $p = 0.368 > 0.05$) (Figure 6A).The colony formation ability was significantly decreased in the DOX (2 μg/mL) group compared with the DOX (0 μg/mL) group (63.15% ± 6.47% versus 18.75% ± 2.88%, $p = 0.00124$, $p < 0.01$); however, it was significantly not altered in the DOX (2 μg/mL) + rLV-Cbf5 group compared with the DOX (0 μg/mL) group (63.15% ± 6.47% versus 58.34% ± 3.47%, $p = 0.0873 > 0.05$) (Figure S5). The sphere formation ability was significantly decreased in the DOX (2 μg/mL) group compared with the DOX (0 μg/mL) group (55.74% ± 6.16% versus 18.81% ± 2.51%, $p = 0.00877 < 0.01$); however, it was significantly not altered in the DOX (2 μg/mL) + rLV-Cbf5 group compared with the DOX (0 μg/mL) group (55.74% ± 6.16% versus 46.19% ± 4.21%, $p = 0.1232 > 0.05$) (Figure S6). The weight of transplanted tumors was significantly decreased in the DOX (2 μg/mL) group compared with the DOX (0 μg/mL) group (0.791 ± 0.0914 versus 0.225 ± 0.069 g, $p = 0.000000138 < 0.01$);

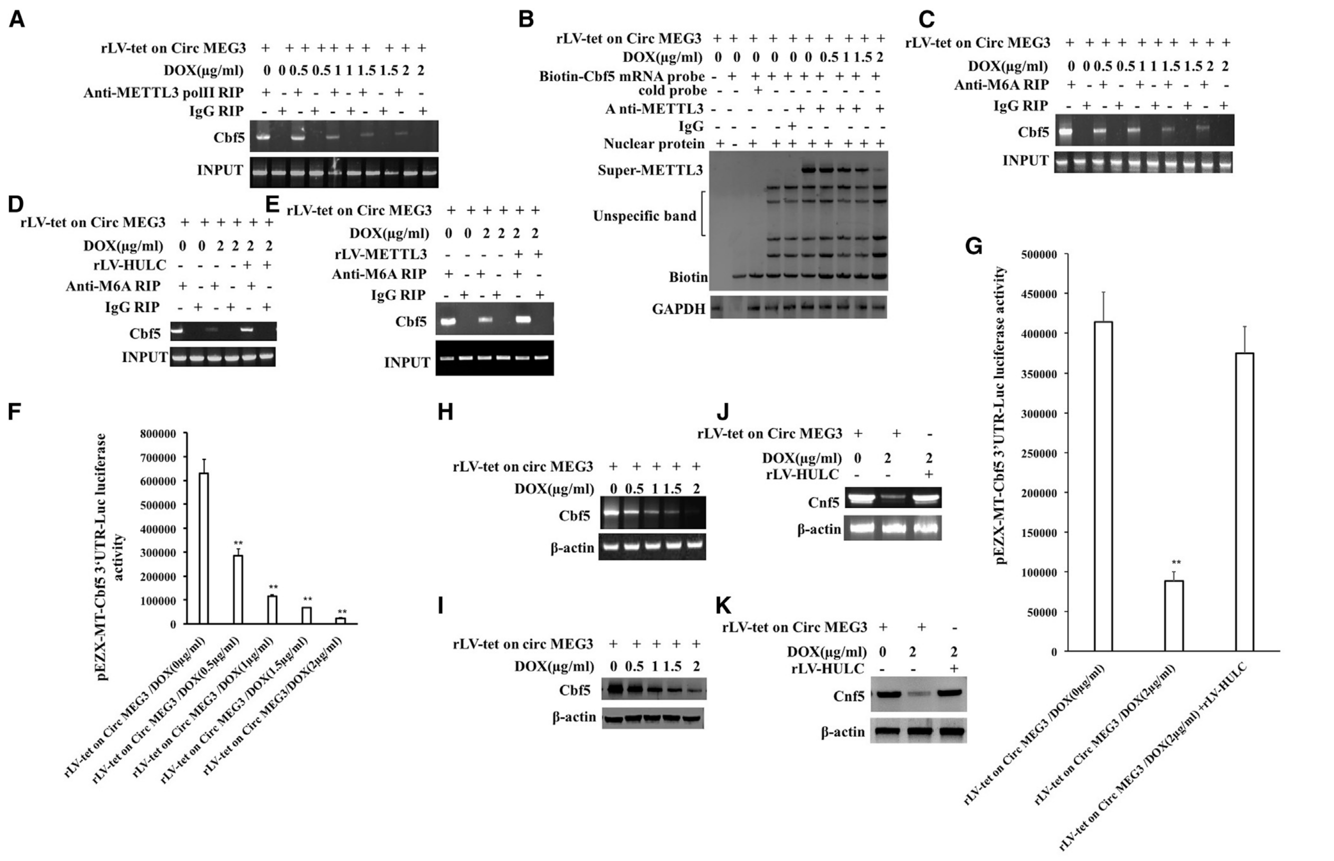

Figure 3. CircMEG3 inhibits the expression of Cbf5 in human liver cancer stem cells

(A) RNA immunoprecipitation (RIP) with anti-METTL3 was performed. IgG RIP was used as a negative control. (B) Super-RNA-protein complex gel migration assay using biotin-labeled Cbf5 mRNA probe and anti-METTL3, anti-Biotin. IgG super-EMSA was a negative control. (C) RIP with anti-M6A was performed. IgG RIP was used as a negative control. (D) RIP with anti-M6A was performed. IgG RIP was used as a negative control. (E) RIP with anti-M6A was performed. IgG RIP was used as a negative control. (F and G) The pEZX-MT-Cbf5 3′ UTR-Luc luciferase reporter plasmid was transfected into these four stable liver cancer stem cell lines, and the activity was detected. Each experiment was repeated three times. $^{**}p < 0.01$, $^{*}p < 0.05$. (H and J) RT-PCR analysis was performed using Cbf5 primers. β-Actin was used as an internal reference gene. (I and K) Western blotting with anti-Cbf5 was performed. β-Actin was an internal reference gene.

however, it was significantly not altered in the DOX (2 μg/mL) + rLV-Cbf5 group compared with the DOX (0 μg/mL) group (0.791 ± 0.0914 versus 0.815 ± 0.136 g, p = 0.18668 > 0.05) (Figures 6B and 6C). The appearance time of transplanted tumors in nude mice was significantly increased in the DOX (2 μg/mL) group compared with the DOX (0 μg/mL) group (7.5 ± 1.19 versus 15.625 ± 1.68 days, p = 0.0000037 < 0.01); however, it was not significantly altered in the DOX (2 μg/mL) + rLV-Cbf5 group compared with the DOX (0 μg/mL) group (7.5 ± 1.19 versus 7.125 ± 1.25 days, p = 0.299 > 0.05). The malignancy of transplanted tumors was significantly decreased in the DOX (2 μg/mL) group compared with the DOX (0 μg/mL) group; however, it was not significantly altered in the DOX (2 μg/mL) + rLV-Cbf5 group compared with the DOX (0 μg/mL) group (Figure 6D). Immunoblotting showed that the PCNA expression was significantly decreased in the DOX (2 μg/mL) group compared with the DOX (0 μg/mL) group; however, it was significantly not altered in the DOX (2 μg/mL) + rLV-Cbf5 group compared with the DOX (0 μg/mL) group (Figure 6E). Immunoblotting showed that the Glypican-3 expression was significantly decreased in the DOX (2 μg/mL) group compared with the DOX (0 μg/mL) group; however, it was significantly not altered in the DOX (2 μg/mL) + rLV-Cbf5 group compared with the DOX (0 μg/mL) group (Figure 6F). Collectively, these results suggest that increased Cbf5-telomerase activity abrogates the ability of CircMEG3 to inhibit malignant differentiation of human liver CSCs.

DISCUSSION

CSCs in hepatocellular carcinoma are able to exclusively initiate tumorigenesis.[34–36] For example, LCSC-related mitochondrial metabolism contributes to the liver CSC features.[37] The Wnt/beta-catenin is believed to play an important role in the pathogenesis of CSC formation.[38] To date, the functions and regulatory mechanisms of CircMEG3 have not fully been elucidated in liver CSCs. We first demonstrate that CircMEG3 inhibits the growth of liver CSCs by inhibiting telomerase activity dependent on HULC and Cbf5 in human liver CSCs (Figure 7).

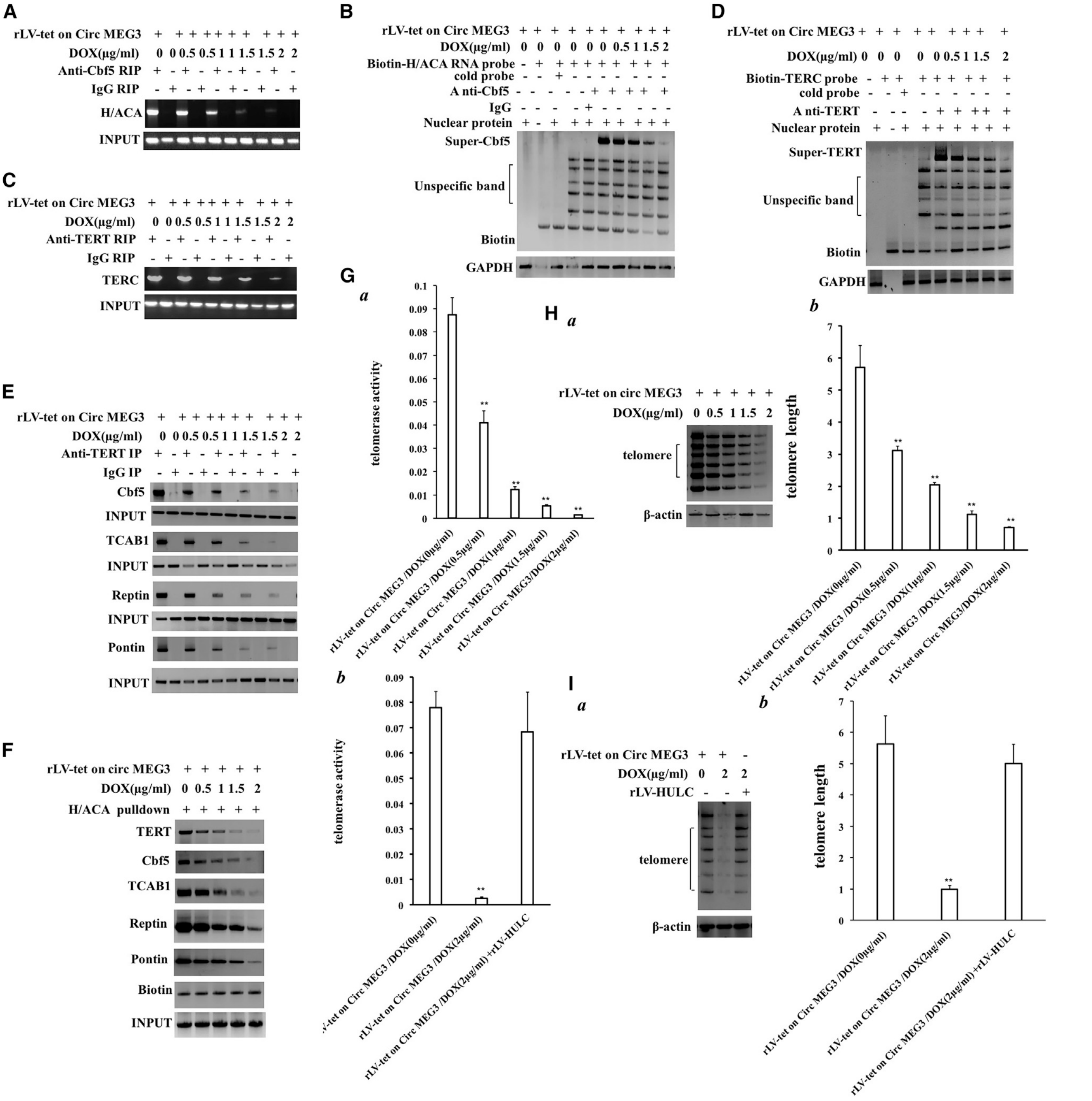

Figure 4. CircMEG3 inhibits telomerase activity and shortens telomere life

(A) RIP with anti-Cbf5 was performed. H/ACA was amplified by RT-PCR. IgG RIP was used as a negative control. (B) Super-RNA-protein complex gel migration assay using biotin-labeled H/ACA mRNA probe and anti-Cbf5, anti-Biotin. IgG super-EMSA was a negative control. (C) RIP with anti-TERT was performed. TERC was amplified by RT-PCR. IgG RIP was used as a negative control. (D) Super-RNA-protein complex gel migration assay using biotin-labeled TERC RNA probe and anti-TERT, anti-Biotin. IgG super-EMSA was a negative control. (E) Co-immunoprecipitation was performed using anti-TERT. IgG co-immunoprecipitation was used as a negative control. (F) The RNA pull-down analysis was performed using biotinylated H/ACA probes and anti-TERT, anti-Cbf5, anti-TCAB1, anti-Reptin, and anti-Pontin. Histone H3 is used as INPUT, and Biotin is used as an internal reference. (Ga and Gb) Telomerase activity was examined by quantitative telomerase activity assay (TRAP). $^{**}p < 0.01$, $^{*}p < 0.05$. (Ha and Ia) The analysis of telomere DNA was analyzed by PCR-Southern blotting. (Hb and Ib) Quantitative PCR amplification of telomere DNA. Each group of values is expressed as mean ± standard deviation (SD; n = 3). $^{**}p < 0.01$, $^{*}p < 0.05$.

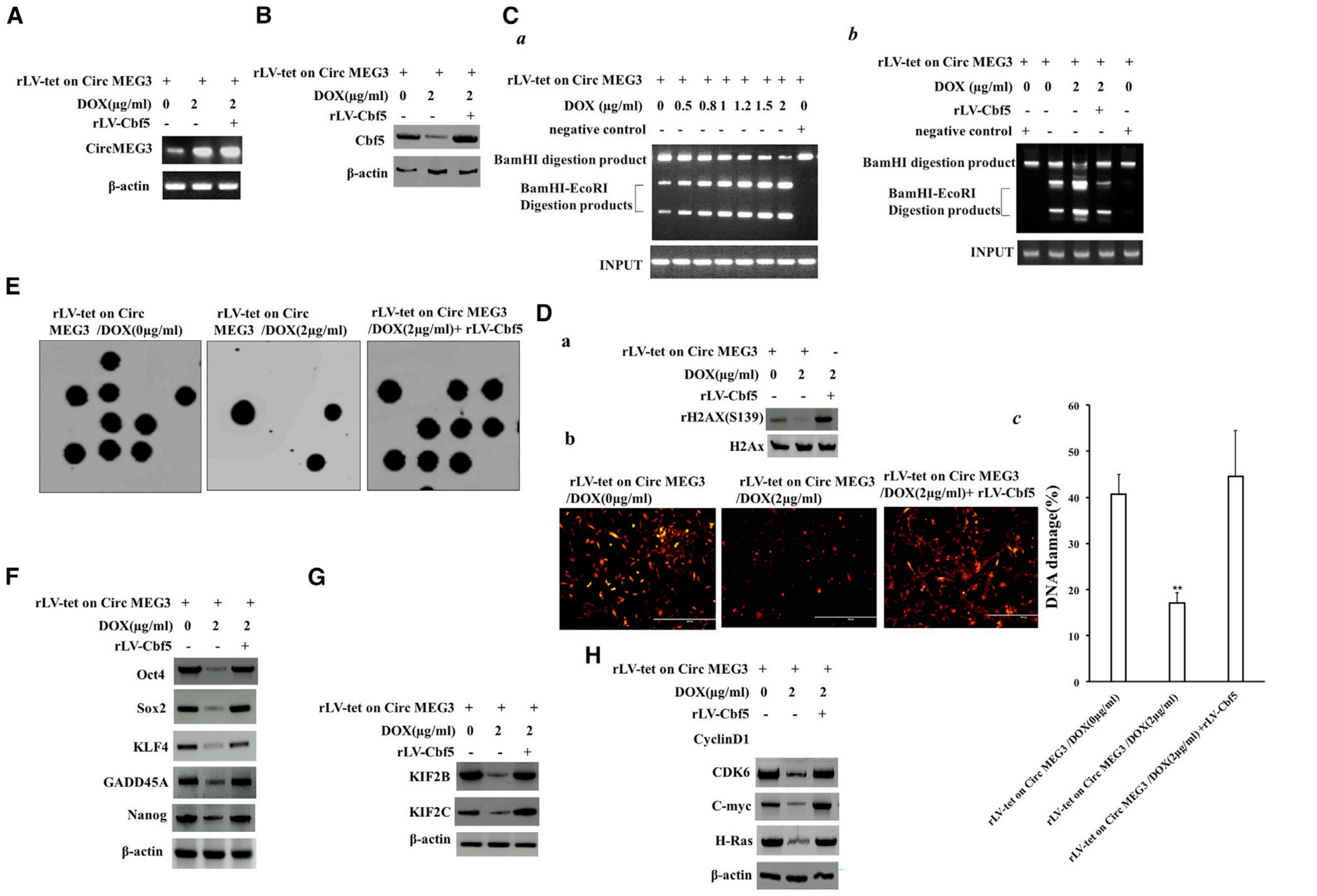

Figure 5. Increased Cbf5-telomerase activity abrogates the functions of CircMEG3 in human liver cancer stem cells

(A) The back-to-back RT-PCR analysis was carried out using CircMEG3 primers in DOX (0 μg/mL), DOX (2 μg/mL), and DOX (2 μg/mL + rLV-Cbf5) groups. β-Actin was used as an internal reference gene. (B) The total protein was subjected to western blotting using anti-Cbf5. β-Actin was an internal reference gene. (Ca and Cb) After cells were transfected with plasmids with mismatch, the restriction endonuclease with BamHI and EcoRI was performed for detecting mismatched plasmid DNA injury repair. (Da) The level of DNA damage marker rH2AX (Ser139) was detected by immunoblotting after induced by alisertib. (Db and Dc) The level of DNA damage marker rH2AX (S139) was detected by immunostaining of DNA damage after induced by alisertib. (E) Microsatellite instability (MSI) analysis through dot blot using various Biotin labeling MSI probes (Biotin-MSIs). (F) Immunoblotting analysis of Oct4, Sox2, KLF4, and Nanog. (G) Immunoblotting analysis of KIF2B and KIF2C. (H) Western blotting with anti-C-myc, anti-CDK4, and anti-H-Ras was performed. β-Actin was an internal reference gene.

It is worth mentioning that our findings in this study provide novel evidence for a suppressor role of CircMEG3 in human liver cancer. This assertion is based on several observations: (1) CircMEG3 is downregulated expression and negatively correlated with the expression of telomerase-related gene Cbf5 in human liver cancer; and (2) CircMEG3 inhibits the growth ability of human liver CSCs *in vitro* and *in vivo*. A MEG3 acts as an antitumor component in different cancer cells, such as breast and liver cancer cells.[39] *MEG3* activated by vitamin D suppresses glycolysis in cancer,[40] and MEG3 induces invasion of glioma cells via autophagy.[41] Moreover, MEG3 promotes differentiation of porcine satellite cells by sponging miR-423-5p[42] and is involved in pituitary tumor invasiveness.[43] In addition, MEG3 inhibits the inflammatory response of ankylosing spondylitis,[44] and MEG3 inhibits HMEC-1 cell growth and migration.[45] Also, MEG3 inhibits the progression of prostate cancer by facilitating H3K27 trimethylation,[46] and MEG3 knockdown attenuates endoplasmic reticulum stress-mediated apoptosis.[47] Furthermore, uric acid enhances autophagy through the MEG3/miR-7-5p/EGFR axis.[48] Interestingly, MEG3 interacts with miR-494 to repress bladder cancer progression through targeting PTEN,[49] and MEG3 binds with miR-27a to promote PHLPP2 protein translation and impairs bladder cancer invasion.[50] In addition, MEG3 inhibits breast cancer growth via upregulating endoplasmic reticulum stress.[51] Our present results are consistent with these reports and provide novel evidence for an active role of CircMEG3 in inhibiting the growth of liver CSCs.

Importantly, our results suggest that CircMEG3 inhibits the expression of m6A methyltransferase METTL3 dependent on HULC in human liver CSCs. METTL3 is implicated in many aspects of tumor progression, including tumorigenesis, proliferation, and invasion,[52] and

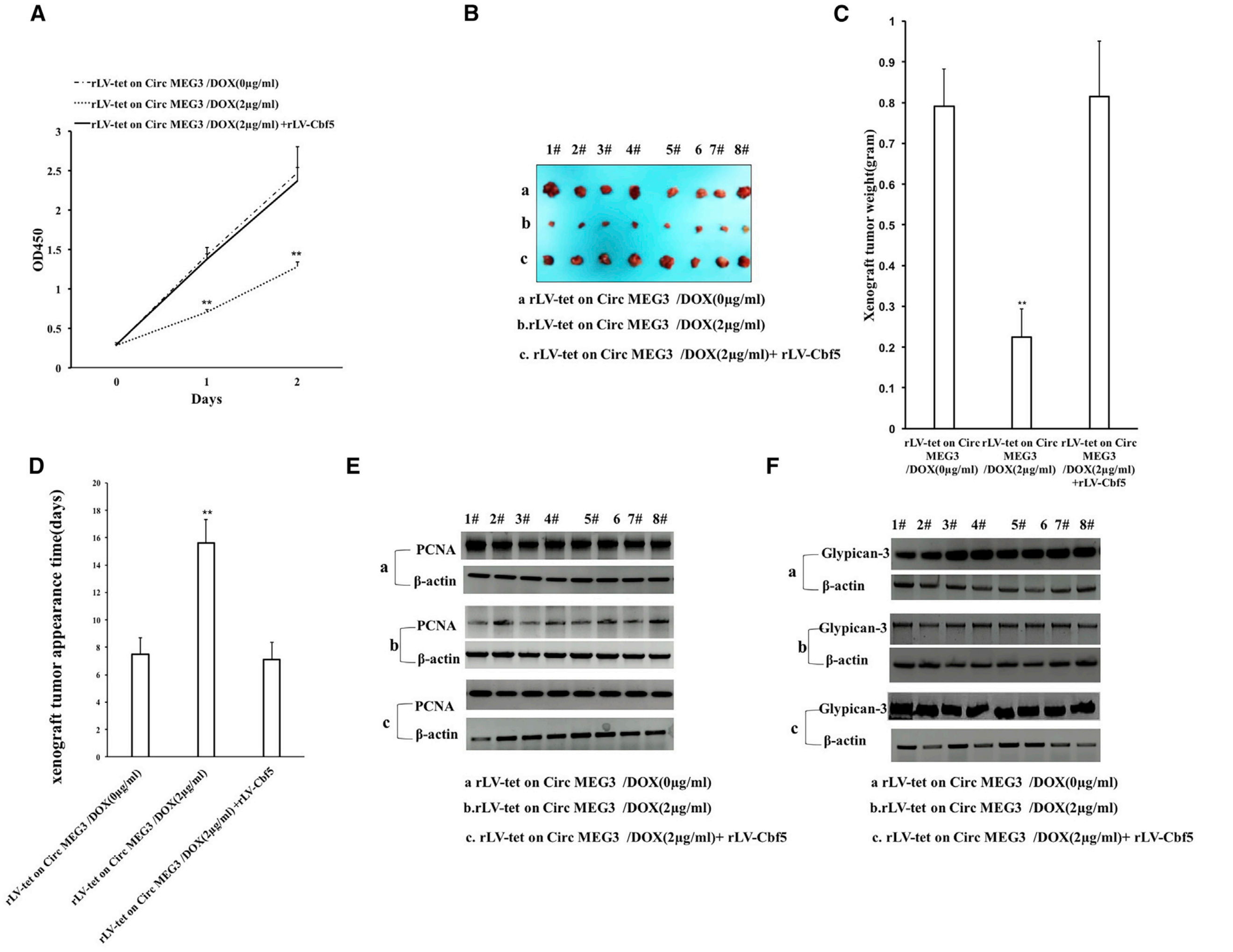

Figure 6. Increased Cbf5-telomerase activity abrogates the ability of CircMEG3 to inhibit malignant differentiation of human liver cancer stem cells

(A) The assay of cell proliferation ability (CCK8). $^{**}p < 0.01$ or $^{*}p < 0.05$ means statistical difference. (B) Photos of transplanted tumors (xenograft) dissected from the axillary of immunocompromised BALB/C nude mice after liver cancer stem cells were inoculated 1 month. (C) Comparison of the size (g) of transplanted tumors in nude mice. (D) Comparison of the appearance time (days) of transplanted tumors in nude mice. $^{**}p < 0.01$ or $^{*}p < 0.05$ means statistical difference is significant. (E and F) Western blot analysis with anti-PCNA. β-Actin was an internal reference gene.

promotes the progression of prostate carcinoma via mediating MYC methylation[53] and enhances cell adhesion through stabilizing integrin β1.[54] Moreover, m6A-dependent glycolysis enhances colorectal cancer progression.[55] Evidentially, our findings provide novel evidence that CircMEG3 inhibits the expression of Cbf5 through METTL3 dependent on HULC in human liver CSCs. A single H/ACA small nucleolar RNA mediates tumor suppression downstream of oncogenic RAS,[56–58] and H/ACA box small nucleolar RNA 7B acts as an oncogene and a potential prognostic biomarker in breast cancer.[59] Our present results are consistent with these reports and provide novel evidence for an active role of CircMEG3 liver CSCs.

Notably, our results suggest that CircMEG3 inhibits telomerase activity dependent on HULC and Cbf5 in human liver CSCs. Studies in telomere-related protein complexes include TRF1, TRF2, Rap1, POT1, TIN2, etc.[60–62] The formation of the T loop of the granules inhibits the ATM-mediated DNA damage response.[63] Telomerase core components include telomerase reverse transcriptase (TERT) and TERC.[64,65] Moreover, telomerase is involved in stem cell self-renewal.[66,67] Studies have shown that mammalian cell telomeres exhibit high levels of histone H3K9me3 and H4K20me3 modifications,[68] and telomeres can rely on RNA polymerase II to generate long-chain ncRNA TERRA.[69,70] It was found that TERRA deletion would result in a reduction in the apparent modification of H3K9me3 at the telomeres.[71] In particular, the 5′-UUAGGG-3′ repeat sequence of TERRA can bind to TERC through base-pairing, which competitively inhibits the activity of telomerase.[72,73] In recent years, new phenomena that regulate cell telomere function have been

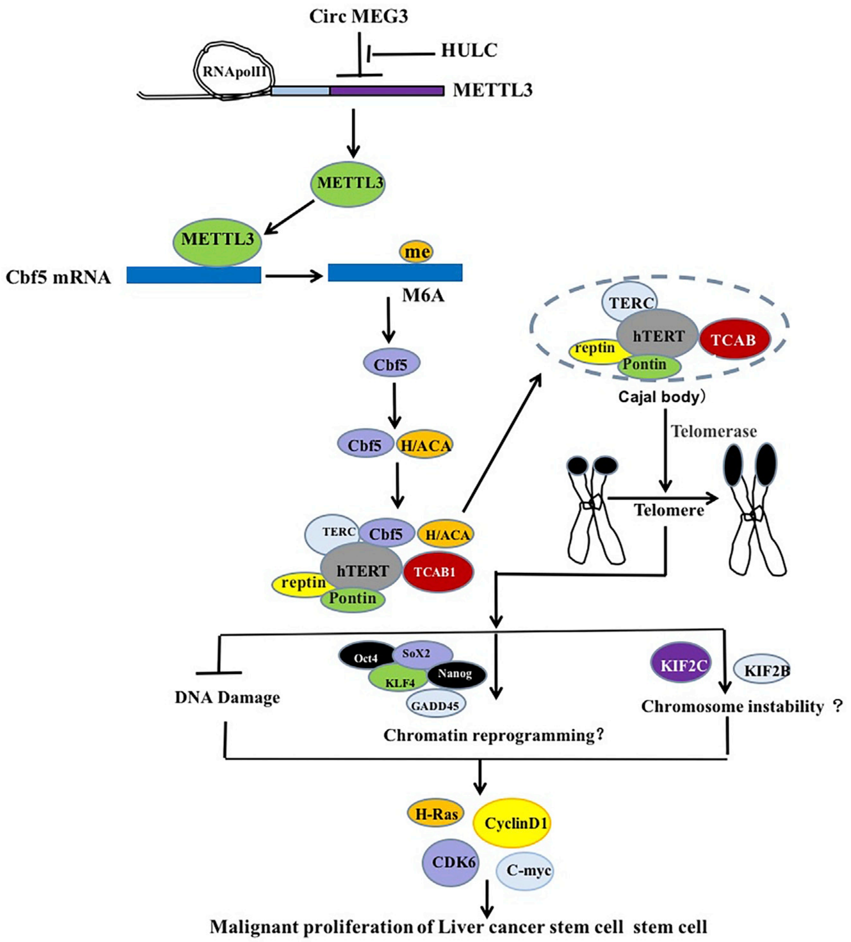

Figure 7. Schematic diagram about molecular mechanism by which CircMEG3 blocks telomere function dependent on Cbf5 and inhibits the malignant differentiation of human liver cancer stem cells

discovered, such as selective extension of telomere through DNA break-induced replication mechanism.[74,75] Telomere shieldin complex mediates P53BP1-dependent DNA repair,[76] and telomere necrosis activates autophagic death.[77,78] What kind of factor can dynamically control telomere remodeling at the spatiotemporal level and control the fate of cells needs to be further investigated.

Furthermore, our study found that CircMEG3 inhibited the expression and function of the telomerase component Cbf5 in human liver CSCs, thereby inhibiting the lifespan of telomeres in liver CSCs. It is worth noting that mutations in the Cbf5 protein in H/ACA RNP that catalyzes the modification of RNA pseudouracil and the synthesis of telomeres will cause shortening of telomeres.[79] Studies have shown that TCAB1 is a component of telomerase, and it plays a role in the nuclear processing of the Cajal body.[80] It has also been found that the TRFH domain of TRF2 regulates the formation of telomere T loops while inhibiting ATM activity.[81] An activity switch in human telomerase was based on RNA conformation and shaped by TCAB1.[82] Minimized human telomerase maintains telomeres and resolves endogenous roles of H/ACA proteins, TCAB1, and Cajal bodies.[83] WRAP53β mediates site-specific interactions between Cajal body factors and DNA repair proteins.[84] Reptin drives tumor progression,[85] and Pontin/Tip49 negatively regulates JNK-mediated cell death.[86]

Strikingly, our studies have found that CircMEG3 can promote DNA damage repair and inhibit DNA instability. CircMEG3 is involved in DNA damage repair and DNA microsatellite instability. Studies have shown that when the genome is damaged by DNA stimulation inside and outside the cell, it may lead to genome instability.[87] Therefore, DNA repair in nucleosomes is essential for gene regulation,[88] and various DNA repair pathways maintain the genome stability.[89] DNA breaks and the activation of the DNA damage response arise from endogenous replication stress.[90] Octamer-binding transcription factor 4 (OCT4) is essential in embryogenesis and pluripotency.[91] SOX2 protein may serve as a novel prognostic factor for colorectal cancer.[92] KLF4 regulates gene expression through transcriptional activation or repression.[93] NANOG is a novel therapeutic target for ovarian cancer (OC),[94] and Kinesin family member 2C aggravates the progression of hepatocellular carcinoma.[95] In addition, Plk1 regulates the kinesin-13 protein Kif2b to promote chromosome segregation,[96] and PCAT-1 plays an oncogenic role in epithelial OC by modulating cyclinD1/CDK4.[97] It was confirmed that our present results are consistent with these reports and provide novel evidence for a suppressor role of CircMEG3 in inhibiting malignant growth of liver cancer.

Another significant finding is that long ncRNA HULC plays an important role for regulating CircMEG3. Our present results are consistent with these reports and provide novel evidence for a suppressor role of CircMEG3 in inhibiting malignant growth of liver cancer via altering HULC. HULC is highly upregulated in hepatocellular carcinoma and in several other cancers.[98] Also, HULC induces the progression of osteosarcoma by regulating the miR-372-3p/HMGB1 signaling axis,[99] and HULC accelerates the growth of human liver CSCs via autophagy.[100] In particular, miR24-2 promotes malignant progression of human liver CSCs dependent on HULC.[101] In addition, H-Ras is a unique isoform of the Ras GTPase family.[102] Moreover, inhibiting the cell-cycle kinases CDK4 and CDK6 results in a significant therapeutic effect in several cancers.[103]

In conclusion, our results suggest that increased Cbf5-telomerase activity abrogates the ability of CircMEG3 to inhibit malignant

differentiation of human liver CSCs. These observations provide important basic information for finding effective liver cancer therapeutic targets. We will further study the exact mechanism of Circ-MEG3 in the development of liver cancer and its clinical application.

MATERIALS AND METHODS

hLCSC sorting

The hLCSCs were isolated from human liver cancer line Huh7 using CD133/CD44/CD24/EpCAM MicroBead Kits (MACS Technology, Miltenyi Biotech, Boston, MA, USA) and MACS Technology operation according to the manufacturer.

RT-PCR

cDNA was prepared by using oligonucleotide (dT), random primers, and First-Strand Synthesis System (Invitrogen). PCR analysis was performed according to the manufacturer. β-Actin was used as an internal control.

Western blotting

Proteins were separated on a 10% sodium dodecyl sulfate-polyacrylamide gel electrophoresis and transferred onto a nitrocellulose membranes (Invitrogen). The blots were incubated with antibody overnight at 4°C. Signals were visualized by enhanced chemiluminescence plus kit (GE Healthcare).

Super-RNA-EMSA

Cells were washed and scraped in ice-cold phosphate-buffered saline (PBS) to prepare nuclei for electrophoretic gel mobility shift assay with the use of the gel shift assay system (Promega) modified according to the manufacturer's instructions.

Chromatin immunoprecipitation (ChIP) assay

Crossed-linked cells were washed with PBS, resuspended in lysis buffer, and sonicated for 10 min in a SONICS VibraCell to generate DNA fragments. Chromatin extracts were pre-cleared with protein A/G-Sepharose beads and immunoprecipitated with specific antibody on protein A/G-Sepharose beads. After washing, elution, and de-cross-linking, the ChIP DNA was measured by PCR.

Telomere length assay

ScienCell's Relative Human Telomere Length Quantification qPCR Assay Kit (RHTLQ) is designed to directly compare the average telomere length of the samples.

Cell colony formation efficiency assay

Cells were incubated in a humidified atmosphere of 5% CO_2 incubator at 37°C for 10 days. For visualization, colonies were stained with 0.5% crystal violet (sigma) in 50% methanol and 10% glacial acetic acid.

Tumorigenesis test *in vivo*

Four-week-old male athymic BALB/c mice were maintained in the Tongji university animal facilities approved by the China Association for accreditation of laboratory animal care. Athymic BALB/c mice were injected with LCSC cells at the armpit area subcutaneously. The mice were then sacrificed and the tumors recovered. A portion of each tumor was fixed in 4% paraformaldehyde and embedded in paraffin for histological examination and immunohistochemical staining.

SUPPLEMENTAL INFORMATION

Supplemental Information can be found online at https://doi.org/10.1016/j.omtn.2020.11.009.

ACKNOWLEDGMENTS

This study was supported by grants from National Natural Science Foundation of China (NCSF No. 81773158, NCSF No. 82073130), Science and Technology Commission of Shanghai Municipality Shanghai Science and Technology Plan Basic Research Field Project (19JC1415200), and Science and Technology Commission of Shanghai Municipality Shanghai Science and Technology Plan Basic Research Field Project (20JC1411400).

AUTHOR CONTRIBUTIONS

D.L. conceived the study and participated in the study design, performance, coordination, and manuscript writing. X.J., L.X., Y.C., R.Q., S.S., Y.L., S.X., L.W., H.P., X.G., T.L., J.X., J.L., and S.J. performed the research. All authors have read and approved the final manuscript.

DECLARATION OF INTERESTS

The authors declare no competing interests.

REFERENCES

1. Plaks, V., Kong, N., and Werb, Z. (2015). The cancer stem cell niche: how essential is the niche in regulating stemness of tumor cells? Cell Stem Cell *16*, 225–238.
2. Wang, Y., He, L., Du, Y., Zhu, P., Huang, G., Luo, J., Yan, X., Ye, B., Li, C., Xia, P., et al. (2015). The long noncoding RNA lncTCF7 promotes self-renewal of human liver cancer stem cells through activation of Wnt signaling. Cell Stem Cell *16*, 413–425.
3. Bahr, C., von Paleske, L., Uslu, V.V., Remeseiro, S., Takayama, N., Ng, S.W., Murison, A., Langenfeld, K., Petretich, M., Scognamiglio, R., et al. (2018). A Myc enhancer cluster regulates normal and leukaemic haematopoietic stem cell hierarchies. Nature *553*, 515–520.
4. Xu, X.L., Xing, B.C., Han, H.B., Zhao, W., Hu, M.H., Xu, Z.L., Li, J.Y., Xie, Y., Gu, J., Wang, Y., and Zhang, Z.Q. (2010). The properties of tumor-initiating cells from a hepatocellular carcinoma patient's primary and recurrent tumor. Carcinogenesis *31*, 167–174.
5. Shi, Y., Lan, F., Matson, C., Mulligan, P., Whetstine, J.R., Cole, P.A., Casero, R.A., and Shi, Y. (2004). Histone Demethylation Mediated by the Nuclear Amine Oxidase Homolog LSD1. Cell *119*, 941–953.
6. Liu, P., Chen, M., Liu, Y., Qi, L.S., and Ding, S. (2018). CRISPR-Based Chromatin Remodeling of the Endogenous Oct4 or Sox2 Locus Enables Reprogramming to Pluripotency. Cell Stem Cell *22*, 252–261.e4.
7. Reinberg, D., and Vales, L.D. Chromatin domains rich in inheritance. Science 361, 33–34.
8. Black, J.B., Adler, A.F., Wang, H.G., D'Ippolito, A.M., Hutchinson, H.A., Reddy, T.E., Pitt, G.S., Leong, K.W., and Gersbach, C.A. (2016). Targeted Epigenetic Remodeling of Endogenous Loci by CRISPR/Cas9-Based Transcriptional Activators Directly Converts Fibroblasts to Neuronal Cells. Cell Stem Cell *19*, 406–414.

9. Ventham, N.T., Kennedy, N.A., Nimmo, E.R., and Satsangi, J. (2013). Beyond gene discovery in inflammatory bowel disease: the emerging role of epigenetics. Gastroenterology *145*, 293–308.

10. Allshire, R.C., and Madhani, H.D. (2018). Ten principles of heterochromatin formation and function. Nat. Rev. Mol. Cell Biol. *19*, 229–244.

11. Ji, C., and Guo, X. (2019). The clinical potential of circulating microRNAs in obesity. Nat. Rev. Endocrinol. *15*, 731–743.

12. Ullah, M., Ng, N.N., Concepcion, W., and Thakor, A.S. (2020). Emerging role of stem cell-derived extracellular microRNAs in age-associated human diseases and in different therapies of longevity. Ageing Res. Rev. *57*, 100979.

13. Rupaimoole, R., and Slack, F.J. (2017). MicroRNA therapeutics: towards a new era for the management of cancer and other diseases. Nat. Rev. Drug Discov. *16*, 203–222.

14. Vishnubalaji, R., Shaath, H., Elango, R., and Alajez, N.M. (2020). Noncoding RNAs as potential mediators of resistance to cancer immunotherapy. Semin. Cancer Biol. *65*, 65–79.

15. Wang, X., Luo, G., Zhang, K., Cao, J., Huang, C., Jiang, T., Liu, B., Su, L., and Qiu, Z. (2018). Hypoxic Tumor-Derived Exosomal miR-301a Mediates M2 Macrophage Polarization via PTEN/PI3Kγ to Promote Pancreatic Cancer Metastasis. Cancer Res. *78*, 4586–4598.

16. Wei, L., Wang, X., Lv, L., Liu, J., Xing, H., Song, Y., Xie, M., Lei, T., Zhang, N., and Yang, M. (2019). The emerging role of microRNAs and long noncoding RNAs in drug resistance of hepatocellular carcinoma. Mol. Cancer *18*, 147.

17. Han, L., Cheng, J., and Li, A. (2020). hsa_circ_0072387 suppresses proliferation, metastasis, and glycolysis of oral squamous cell carcinoma cells by downregulating miR-503-5p. Cancer Biother. Radiopharm. Published online April 17, 2020. https://doi.org/10.1089/cbr.2019.3371.

18. Xu, Y.J., Yu, H., and Liu, G.X. (2020). Hsa_circ_0031288/hsa-miR-139-3p/Bcl-6 regulatory feedback circuit influences the invasion and migration of cervical cancer HeLa cells. J. Cell. Biochem. *121*, 4251–4260.

19. Cui, S., and Zhang, L. (2020). circ_001653 Silencing Promotes the Proliferation and ECM Synthesis of NPCs in IDD by Downregulating miR-486-3p-Mediated CEMIP. Mol. Ther. Nucleic Acids *20*, 385–399.

20. Zhang, Q., Miao, Y., Fu, Q., Hu, H., Chen, H., Zeng, A., Jin, Y., Jiang, Y., Qian, L., Wu, L., et al. (2020). CircRNACCDC66 regulates cisplatin resistance in gastric cancer via the miR-618/BCL2 axis. Biochem. Biophys. Res. Commun. *526*, 713–720.

21. Yang, L., Liang, H., Meng, X., Shen, L., Guan, Z., Hei, B., Yu, H., Qi, S., and Wen, X. (2020). mmu_circ0000790 is involved in pulmonary vascular remodeling in mice with HPH via microRNA-374c-mediated FOXC1. Mol. Ther. Nucleic Acids *20*, 292–307.

22. Cao, L., Wang, M., Dong, Y., Xu, B., Chen, J., Ding, Y., Qiu, S., Li, L., Karamfilova Zaharieva, E., Zhou, X., and Xu, Y. (2020). Circular RNA circRNF20 promotes breast cancer tumorigenesis and Warburg effect through miR-487a/HIF-1α/HK2. Cell Death Dis. *11*, 145.

23. Sartorius, K., Makarova, J., Sartorius, B., An, P., Winkler, C., Chuturgoon, A., and Kramvis, A. (2019). The Regulatory Role of MicroRNA in Hepatitis-B Virus-Associated Hepatocellular Carcinoma (HBV-HCC) Pathogenesis. Cells *8*, 1504.

24. Nio, K., Yamashita, T., and Kaneko, S. (2017). The evolving concept of liver cancer stem cells. Mol. Cancer *16*, 4.

25. Zhou, B., Guo, H., and Tang, J. (2019). Long Non-Coding RNA TFAP2A-AS1 Inhibits Cell Proliferation and Invasion in Breast Cancer via miR-933/SMAD2. Med. Sci. Monit. *25*, 1242–1253.

26. Zhu, X., Shao, P., Tang, Y., Shu, M., Hu, W.W., and Zhang, Y. (2019). hsa_circRNA_100533 regulates GNAS by sponging hsa_miR_933 to prevent oral squamous cell carcinoma. J. Cell. Biochem. *120*, 19159–19171.

27. Poliseno, L., Haimovic, A., Segura, M.F., Hanniford, D., Christos, P.J., Darvishian, F., Wang, J., Shapiro, R.L., Pavlick, A.C., Berman, R.S., et al. (2012). Histology-specific microRNA alterations in melanoma. J. Invest. Dermatol. *132*, 1860–1868.

28. Wu, C., Zhao, Y., Liu, Y., Yang, X., Yan, M., Min, Y., Pan, Z., Qiu, S., Xia, S., Yu, J., et al. (2018). Identifying miRNA-mRNA regulation network of major depressive disorder in ovarian cancer patients. Oncol. Lett. *16*, 5375–5382.

29. di Gennaro, A., Damiano, V., Brisotto, G., Armellin, M., Perin, T., Zucchetto, A., Guardascione, M., Spaink, H.P., Doglioni, C., Snaar-Jagalska, B.E., et al. (2018). A p53/miR-30a/ZEB2 axis controls triple negative breast cancer aggressiveness. Cell Death Differ. *25*, 2165–2180.

30. Wang, T., Chen, G., Ma, X., Yang, Y., Chen, Y., Peng, Y., Bai, Z., Zhang, Z., Pei, H., and Guo, W. (2019). MiR-30a regulates cancer cell response to chemotherapy through SNAI1/IRS1/AKT pathway. Cell Death Dis. *10*, 153.

31. Zhu, J., Zeng, Y., Li, W., Qin, H., Lei, Z., Shen, D., Gu, D., Huang, J.A., and Liu, Z. (2017). CD73/NT5E is a target of miR-30a-5p and plays an important role in the pathogenesis of non-small cell lung cancer. Mol. Cancer *16*, 34.

32. Donato, R., Sorci, G., and Giambanco, I. (2017). S100A6 protein: functional roles. Cell. Mol. Life Sci. *74*, 2749–2760.

33. Ohuchida, K., Mizumoto, K., Ishikawa, N., Fujii, K., Konomi, H., Nagai, E., and Yamaguchi, K. (2005). The role of S100A6 in pancreatic cancer development and its clinical implication as a diagnostic marker and therapeutic target. Clin. Cancer Res. *11*, 7785–7793.

34. Schulte, L.A., López-Gil, J.C., Sainz, B., Jr., and Hermann, P.C. (2020). The Cancer Stem Cell in Hepatocellular Carcinoma. Cancers (Basel) *12*, E684.

35. Sukowati, C.H.C. (2019). Heterogeneity of Hepatic Cancer Stem Cells. Adv. Exp. Med. Biol. *1139*, 59–81.

36. Wu, H.J., and Chu, P.Y. (2019). Role of Cancer Stem Cells in Cholangiocarcinoma and Therapeutic Implications. Int. J. Mol. Sci. *20*, 4154.

37. Chang, C.W., Lo, J.F., and Wang, X.W. (2019). Roles of mitochondria in liver cancer stem cells. Differentiation *107*, 35–41.

38. Mavila, N., and Thundimadathil, J. (2019). The Emerging Roles of Cancer Stem Cells and Wnt/Beta-Catenin Signaling in Hepatoblastoma. Cancers (Basel) *11*, E1406.

39. Al-Rugeebah, A., Alanazi, M., and Parine, N.R. (2019). MEG3: an Oncogenic Long Non-coding RNA in Different Cancers. Pathol. Oncol. Res. *25*, 859–874.

40. Zuo, S., Wu, L., Wang, Y., and Yuan, X. (2020). Long non-coding RNA MEG3 Activated by vitamin D suppresses glycolysis in colorectal cancer via Promoting c-Myc degradation. Front. Oncol. *10*, 274.

41. Yang, Z., Bian, E., Xu, Y., Ji, X., Tang, F., Ma, C., Wang, H., and Zhao, B. (2020). Meg3 Induces EMT and Invasion of Glioma Cells via Autophagy. OncoTargets Ther. *13*, 989–1000.

42. Cheng, X., Li, L., Shi, G., Chen, L., Fang, C., Li, M., and Li, C. (2020). MEG3 promotes differentiation of porcine satellite cells by sponging miR-423-5p to relieve inhibiting effect on SRF. Cells *9*, E449.

43. Zhu, D., Xiao, Z., Wang, Z., Hu, B., Duan, C., Zhu, Z., Gao, N., Zhu, Y., and Wang, H. (2020). MEG3/MIR-376B-3P/HMGA2 axis is involved in pituitary tumor invasiveness. J. Neurosurg. *2020*, 1–13.

44. Li, Y., Zhang, S., Zhang, C., and Wang, M. (2020). LncRNA MEG3 inhibits the inflammatory response of ankylosing spondylitis by targeting miR-146a. Mol. Cell. Biochem. *466*, 17–24.

45. Xu, D., Liu, T., He, L., Han, D., Ma, Y., and Du, J. (2020). LncRNA MEG3 inhibits HMEC-1 cells growth, migration and tube formation via sponging miR-147. Biol. Chem. *401*, 601–615.

46. Zhou, Y., Yang, H., Xia, W., Cui, L., Xu, R., Lu, H., Xue, D., Tian, Z., Ding, T., Cao, Y., et al. (2020). LncRNA MEG3 inhibits the progression of prostate cancer by facilitating H3K27 trimethylation of EN2 through binding to EZH2. J. Biochem. *167*, 295–301.

47. Li, X., Zhao, J., Geng, J., Chen, F., Wei, Z., Liu, C., Zhang, X., Li, Q., Zhang, J., Gao, L., et al. (2019). Long non-coding RNA MEG3 knockdown attenuates endoplasmic reticulum stress-mediated apoptosis by targeting p53 following myocardial infarction. J. Cell. Mol. Med. *23*, 8369–8380.

48. Cao, Y., Wen, J., Li, Y., Chen, W., Wu, Y., Li, J., and Huang, G. (2019). Uric acid and sphingomyelin enhance autophagy in iPS cell-originated cardiomyocytes through lncRNA MEG3/miR-7-5p/EGFR axis. Artif. Cells Nanomed. Biotechnol. *47*, 3774–3785.

49. Shan, G., Tang, T., Xia, Y., and Qian, H.J. (2020). MEG3 interacted with miR-494 to repress bladder cancer progression through targeting PTEN. J. Cell. Physiol. *235*, 1120–1128.

50. Huang, C., Liao, X., Jin, H., Xie, F., Zheng, F., Li, J., Zhou, C., Jiang, G., Wu, X.R., and Huang, C. (2019). MEG3, as a Competing Endogenous RNA, Binds with miR-27a to Promote PHLPP2 Protein Translation and Impairs Bladder Cancer Invasion. Mol. Ther. Nucleic Acids *16*, 51–62.

51. Zhang, Y., Wu, J., Jing, H., Huang, G., Sun, Z., and Xu, S. (2019). Long noncoding RNA MEG3 inhibits breast cancer growth via upregulating endoplasmic reticulum stress and activating NF-κB and p53. J. Cell. Biochem. *120*, 6789–6797.

52. Zheng, W., Dong, X., Zhao, Y., Wang, S., Jiang, H., Zhang, M., Zheng, X., and Gu, M. (2019). Multiple Functions and Mechanisms Underlying the Role of METTL3 in Human Cancers. Front. Oncol. *9*, 1403.

53. Yuan, Y., Du, Y., Wang, L., and Liu, X. (2020). The M6A methyltransferase METTL3 promotes the development and progression of prostate carcinoma via mediating MYC methylation. J. Cancer *11*, 3588–3595.

54. Li, E., Wei, B., Wang, X., and Kang, R. (2020). METTL3 enhances cell adhesion through stabilizing integrin β1 mRNA via an m6A-HuR-dependent mechanism in prostatic carcinoma. Am. J. Cancer Res. *10*, 1012–1025.

55. Shen, C., Xuan, B., Yan, T., Ma, Y., Xu, P., Tian, X., Zhang, X., Cao, Y., Ma, D., Zhu, X., et al. (2020). m^6A-dependent glycolysis enhances colorectal cancer progression. Mol. Cancer *19*, 72.

56. De Zoysa, M.D., and Yu, Y.T. (2017). Posttranscriptional RNA Pseudouridylation. Enzymes *41*, 151–167.

57. Majumder, M., Mukhopadhyay, S., Kharel, P., and Gupta, R. (2020). The presence of the ACA box in archaeal H/ACA guide RNAs promotes atypical pseudouridylation. RNA *26*, 396–418.

58. McMahon, M., Contreras, A., Holm, M., Uechi, T., Forester, C.M., Pang, X., Jackson, C., Calvert, M.E., Chen, B., Quigley, D.A., et al. (2019). A single H/ACA small nucleolar RNA mediates tumor suppression downstream of oncogenic RAS. eLife *8*, e48847.

59. Sun, Y., Chen, E., Li, Y., Ye, D., Cai, Y., Wang, Q., Li, Q., and Zhang, X. (2019). H/ACA box small nucleolar RNA 7B acts as an oncogene and a potential prognostic biomarker in breast cancer. Cancer Cell Int. *19*, 125.

60. Blackburn, E.H. (1991). Structure and function of telomeres. Nature *350*, 569–573.

61. Palm, W., and de Lange, T. (2008). How shelterin protects mammalian telomeres. Annu. Rev. Genet. *42*, 301–334.

62. Porreca, R.M., Herrera-Moyano, E., Skourti, E., Law, P.P., Gonzalez Franco, R., Montoya, A., Faull, P., Kramer, H., and Vannier, J.B. (2020). TRF1 averts chromatin remodelling, recombination and replication dependent-break induced replication at mouse telomeres. eLife *9*, e49817.

63. Galati, A., Micheli, E., Alicata, C., Ingegnere, T., Cicconi, A., Pusch, M.C., Giraud-Panis, M.J., Gilson, E., and Cacchione, S. (2015). TRF1 and TRF2 binding to telomeres is modulated by nucleosomal organization. Nucleic Acids Res. *43*, 5824–5837.

64. Stone, M.D. (2018). Detailed view of human telomerase enzyme invites rethink of its structure. Nature *557*, 174–175.

65. Blackburn, E.H.E.E., Epel, E.S., and Lin, J. (2015). Human telomere biology: A contributory and interactive factor in aging, disease risks, and protection. Science *350*, 1193–1198.

66. Shay, J.W., and Bacchetti, S. (1997). A survey of telomerase activity in human cancer. Eur. J. Cancer *33*, 787–791.

67. Wang, S., Pike, A.M., Lee, S.S., Strong, M.A., Connelly, C.J., and Greider, C.W. (2017). BRD4 inhibitors block telomere elongation. Nucleic Acids Res. *45*, 8403–8410.

68. Benetti, R., García-Cao, M., and Blasco, M.A. (2007). Telomere length regulates the epigenetic status of mammalian telomeres and subtelomeres. Nat. Genet. *39*, 243–250.

69. Azzalin, C.M., Reichenbach, P., Khoriauli, L., Giulotto, E., and Lingner, J. (2007). Telomeric repeat containing RNA and RNA surveillance factors at mammalian chromosome ends. Science *318*, 798–801.

70. Cusanelli, E., and Chartrand, P. (2015). Telomeric repeat-containing RNA TERRA: a noncoding RNA connecting telomere biology to genome integrity. Front. Genet. *6*, 143.

71. Deng, Z., Norseen, J., Wiedmer, A., Riethman, H., and Lieberman, P.M. (2009). TERRA RNA binding to TRF2 facilitates heterochromatin formation and ORC recruitment at telomeres. Mol. Cell *35*, 403–413.

72. Redon, S., Reichenbach, P., and Lingner, J. (2010). The non-coding RNA TERRA is a natural ligand and direct inhibitor of human telomerase. Nucleic Acids Res. *38*, 5797–5806.

73. Redon, S., Zemp, I., and Lingner, J. (2013). A three-state model for the regulation of telomerase by TERRA and hnRNPA1. Nucleic Acids Res. *41*, 9117–9128.

74. Zhang, J.M., Yadav, T., Ouyang, J., Lan, L., and Zou, L. (2019). Alternative Lengthening of Telomeres through Two Distinct Break-Induced Replication Pathways. Cell Rep. *26*, 955–968.e3.

75. Dilley, R.L., Verma, P., Cho, N.W., Winters, H.D., Wondisford, A.R., and Greenberg, R.A. (2016). Break-induced telomere synthesis underlies alternative telomere maintenance. Nature *539*, 54–58.

76. Noordermeer, S.M., Adam, S., Setiaputra, D., Barazas, M., Pettitt, S.J., Ling, A.K., Olivieri, M., Álvarez-Quilón, A., Moatti, N., Zimmermann, M., et al. (2018). The shieldin complex mediates 53BP1-dependent DNA repair. Nature *560*, 117–121.

77. Zlotorynski, E. (2019). Telomere crisis activates autophagic death. Nat. Rev. Mol. Cell Biol. *20*, 133.

78. Cubiles, M.D., Barroso, S., Vaquero-Sedas, M.I., Enguix, A., Aguilera, A., and Vega-Palas, M.A. (2018). Epigenetic features of human telomeres. Nucleic Acids Res. *46*, 2347–2355.

79. Li, S., Duan, J., Li, D., Ma, S., and Ye, K. (2011). Structure of the Shq1-Cbf5-Nop10-Gar1 complex and implications for H/ACA RNP biogenesis and dyskeratosis congenita. EMBO J. *30*, 5010–5020.

80. Sarek, G., Kotsantis, P., Ruis, P., Van Ly, D., Margalef, P., Borel, V., Zheng, X.F., Flynn, H.R., Snijders, A.P., Chowdhury, D., et al. (2019). CDK phosphorylation of TRF2 controls t-loop dynamics during the cell cycle. Nature *575*, 523–527.

81. Van Ly, D., Low, R.R.J., Frölich, S., Bartolec, T.K., Kafer, G.R., Pickett, H.A., Gaus, K., and Cesare, A.J. (2018). Telomere Loop Dynamics in Chromosome End Protection. Mol. Cell *71*, 510–525.e6.

82. Chen, L., Roake, C.M., Freund, A., Batista, P.J., Tian, S., Yin, Y.A., Gajera, C.R., Lin, S., Lee, B., Pech, M.F., et al. (2018). An Activity Switch in Human Telomerase Based on RNA Conformation and Shaped by TCAB1. Cell *174*, 218–230.e13.

83. Vogan, J.M., Zhang, X., Youmans, D.T., Regalado, S.G., Johnson, J.Z., Hockemeyer, D., and Collins, K. (2016). Minimized human telomerase maintains telomeres and resolves endogenous roles of H/ACA proteins, TCAB1, and Cajal bodies. eLife *5*, e18221.

84. Henriksson, S., and Farnebo, M. (2015). On the road with WRAP53β: guardian of Cajal bodies and genome integrity. Front. Genet. *6*, 91.

85. Mikesch, J.H., Schwammbach, D., Hartmann, W., Schmidt, L.H., Schliemann, C., Angenendt, L., Wiewrodt, R., Marra, A., Thoennissen, N.H., Wardelmann, E., et al. (2018). Reptin drives tumour progression and resistance to chemotherapy in nonsmall cell lung cancer. Eur. Respir. J. *52*, 170163.

86. Wang, X., Huang, X., Wu, C., and Xue, L. (2018). Pontin/Tip49 negatively regulates JNK-mediated cell death in Drosophila. Cell Death Discov. *4*, 8.

87. Williamson, A.K., Zhu, Z., and Yuan, Z.M. (2018). Epigenetic mechanisms behind cellular sensitivity to DNA damage. Cell Stress *2*, 176–180.

88. Matsumoto, S., Cavadini, S., Bunker, R.D., Grand, R.S., Potenza, A., Rabl, J., Yamamoto, J., Schenk, A.D., Schübeler, D., Iwai, S., et al. (2019). DNA damage detection in nucleosomes involves DNA register shifting. Nature *571*, 79–84.

89. Feng, J.X., and Riddle, N.C. (2020). Epigenetics and genome stability. Mamm. Genome *31*, 181–195.

90. Ragu, S., Matos-Rodrigues, G., and Lopez, B.S. (2020). Replication Stress, DNA Damage, Inflammatory Cytokines and Innate Immune Response. Genes (Basel) *11*, E409.

91. Mohiuddin, I.S., Wei, S.J., and Kang, M.H. (2020). Role of OCT4 in cancer stem-like cells and chemotherapy resistance. Biochim. Biophys. Acta Mol. Basis Dis. *1866*, 165432.

92. Zhang, X.H., Wang, W., Wang, Y.Q., Zhu, L., and Ma, L. (2020). The association of SOX2 with clinical features and prognosis in colorectal cancer: A meta-analysis. Pathol. Res. Pract. *216*, 152769.

93. Park, C.S., Lewis, A., Chen, T., and Lacorazza, D. (2019). Concise Review: Regulation of Self-Renewal in Normal and Malignant Hematopoietic Stem Cells by Krüppel-Like Factor 4. Stem Cells Transl. Med. *8*, 568–574.

94. Mahalaxmi, I., Devi, S.M., Kaavya, J., Arul, N., Balachandar, V., and Santhy, K.S. (2019). New insight into NANOG: A novel therapeutic target for ovarian cancer (OC). Eur. J. Pharmacol. *852*, 51–57.

95. Zhang, G.P., Shen, S.L., Yu, Y., Yue, X., Hu, W.J., and Li, S.Q. (2020). Kinesin family member 2C aggravates the progression of hepatocellular carcinoma and interacts with competing endogenous RNA. J. Cell. Biochem. *121*, 4419–4430.

96. Hood, E.A., Kettenbach, A.N., Gerber, S.A., and Compton, D.A. (2012). Plk1 regulates the kinesin-13 protein Kif2b to promote faithful chromosome segregation. Mol. Biol. Cell *23*, 2264–2274.

97. Ding, C., Wei, R., Rodríguez, R.A., and Del Mar Requena Mullor, M. (2019). LncRNA PCAT-1 plays an oncogenic role in epithelial ovarian cancer by modulating cyclinD1/CDK4 expression. Int. J. Clin. Exp. Pathol. *12*, 2148–2156.

98. Klec, C., Gutschner, T., Panzitt, K., and Pichler, M. (2019). Involvement of long non-coding RNA HULC (highly up-regulated in liver cancer) in pathogenesis and implications for therapeutic intervention. Expert Opin. Ther. Targets *23*, 177–186.

99. Li, Y., Liu, J.J., Zhou, J.H., Chen, R., and Cen, C.Q. (2020). LncRNA HULC induces the progression of osteosarcoma by regulating the miR-372-3p/HMGB1 signalling axis. Mol. Med. *26*, 26.

100. Wang, C., Jiang, X., Li, X., Song, S., Meng, Q., Wang, L., Lu, Y., Xin, X., Pu, H., Gui, X., et al. (2020). Long noncoding RNA HULC accelerates the growth of human liver cancer stem cells by upregulating CyclinD1 through miR675-PKM2 pathway via autophagy. Stem Cell Res. Ther. *11*, 8.

101. Wang, L., Li, X., Zhang, W., Yang, Y., Meng, Q., Wang, C., Xin, X., Jiang, X., Song, S., Lu, Y., et al. (2020). miR24-2 Promotes Malignant Progression of Human Liver Cancer Stem Cells by Enhancing Tyrosine Kinase Src Epigenetically. Mol. Ther. *28*, 572–586.

102. Shu, L., Wang, D., Saba, N.F., and Chen, Z.G. (2020). A Historic Perspective and Overview of H-Ras Structure, Oncogenicity, and Targeting. Mol. Cancer Ther. *19*, 999–1007.

103. Álvarez-Fernández, M., and Malumbres, M. (2020). Mechanisms of Sensitivity and Resistance to CDK4/6 Inhibition. Cancer Cell *37*, 514–529.

OMTN, Volume *23*

Supplemental Information

CircMEG3 inhibits telomerase activity by reducing Cbf5 in human liver cancer stem cells

Xiaoxue Jiang, Libo Xing, Yingjie Chen, Rushi Qin, Shuting Song, Yanan Lu, Sijie Xie, Liyan Wang, Hu Pu, Xin Gui, Tianming Li, Jie Xu, Jiao Li, Song Jia, and Dongdong Lu

Supplemental Figure

FigureS1

A

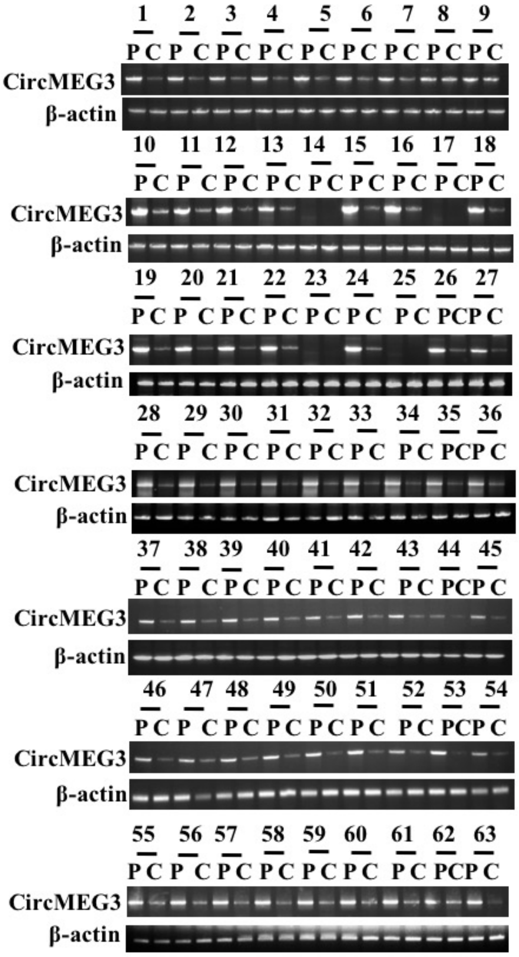

B

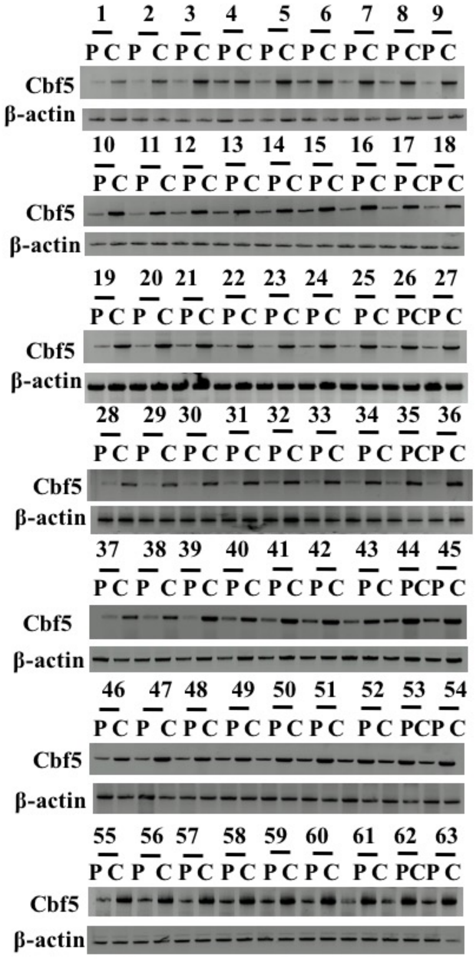

C

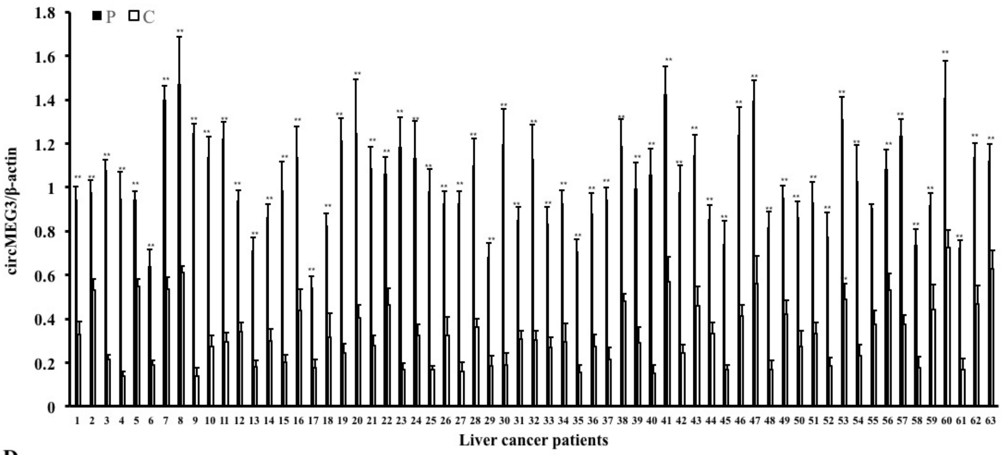

D

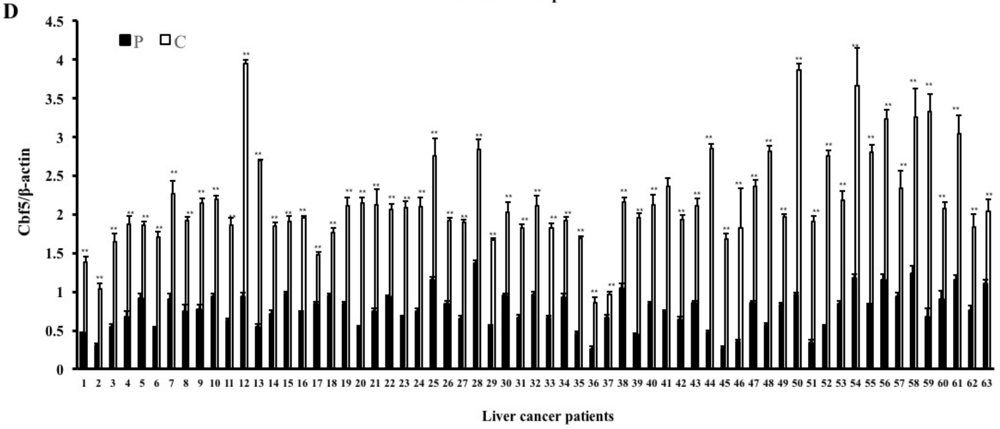

E

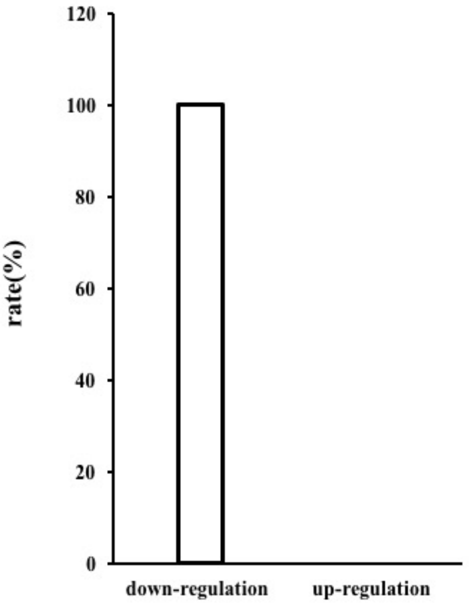

Cir c MEG3 expression in 63 cases of liver cancers

F

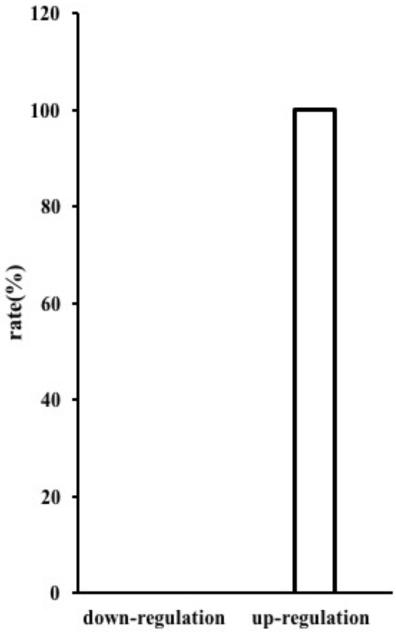

Cbff5 expression in 63 cases of liver cancers

FigureS1. Expression analysis of Circ MEG3 and telomerase-related gene Cbf5 in human liver cancer tissue. A. back-to-back RT-PCR analysis of CircMEG3 expression in liver cancer and its adjacent tissues. β-actin serves as an internal reference. B. Immunoblotting analysis of Cbf5 expression in liver cancer and its adjacent tissues. β-actin serves as an internal reference. C. Real-time back-to-back RT-PCR analysis of Circ MEG3 expression in liver cancer and its adjacent tissues. β-actin serves as an internal reference. D. Real-time RT-PCR analysis of Cbf5 mRNA in liver cancer and its adjacent tissues. β-actin serves as an internal reference.*C*: liver cancer tissues, *P* : adjacent noncancerous tissues. E. Circ MEG3 expression rate in liver cancer and its adjacent tissues. F. Cbf5 expression rate in liver cancer and its adjacent tissues.

FigureS2

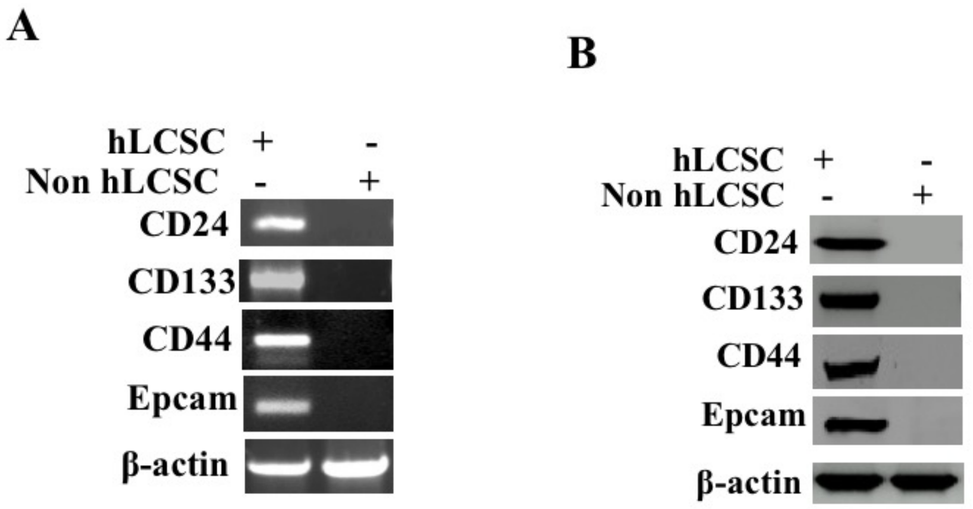

Figure S2 The identification of human liver cancer stem cells. RT-PCR (A) and Western blotting (B) analysis of CD133, CD44, CD24 and EpCAM. β-actin serves as an internal reference..

FigureS3

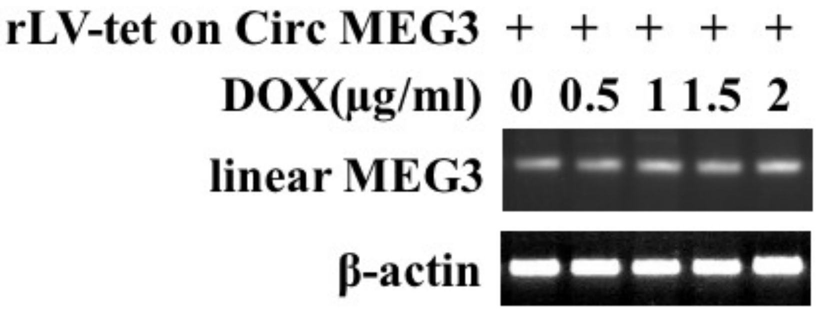

FigureS3 RT-PCR is used to detect linear MEG3 in human live cancer stem cell lines (hLCSCs) infected with rLV-tet on-CircMEG3 at different concentrations of DOX (0μg / ml, 0.5μg / ml, 1μg / ml, 1.5μg / ml, 2μg / ml) (E. semi-quantitative; F. quantitative). β-actin serves as an internal reference.

Figure S4

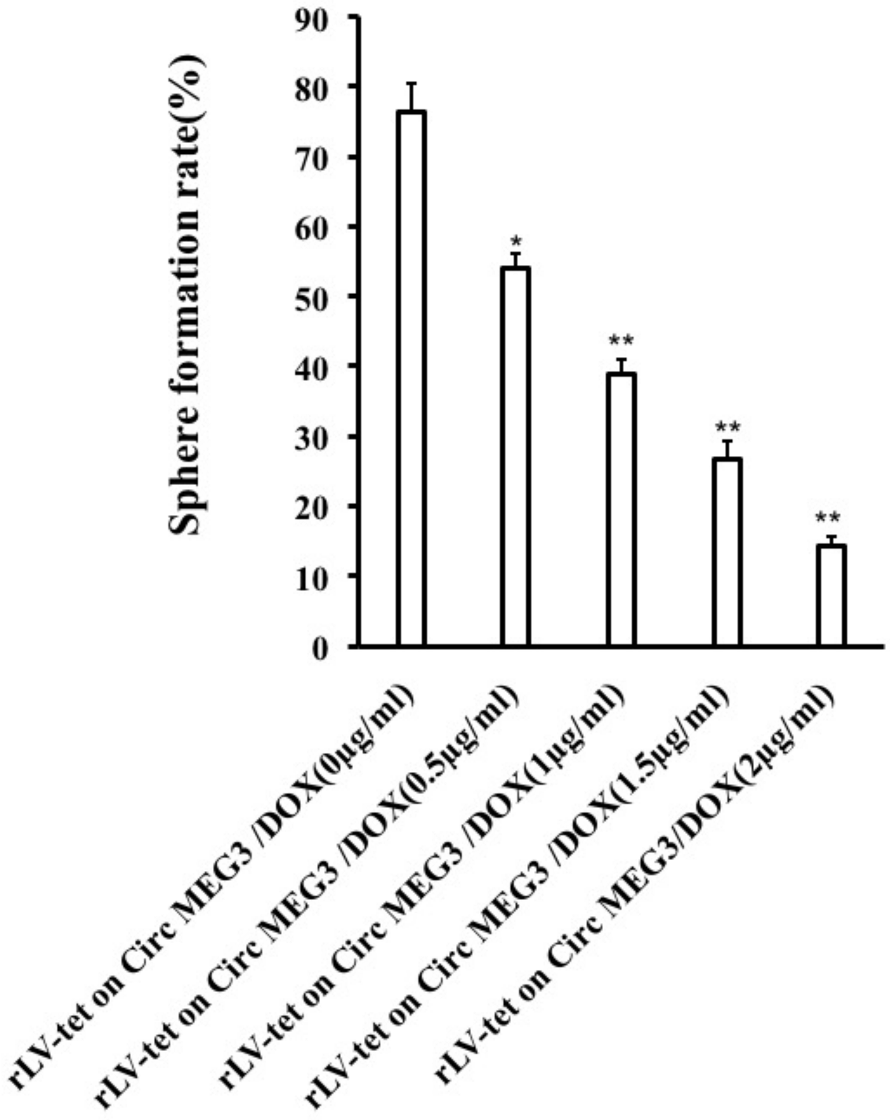

Figure S4 The analysis of human liver cancer stem cell sphere formation ability. The analysis of sphere formation rate. **, P <0.01 or *, P <0.05 means statistical difference is significant.

FigureS5

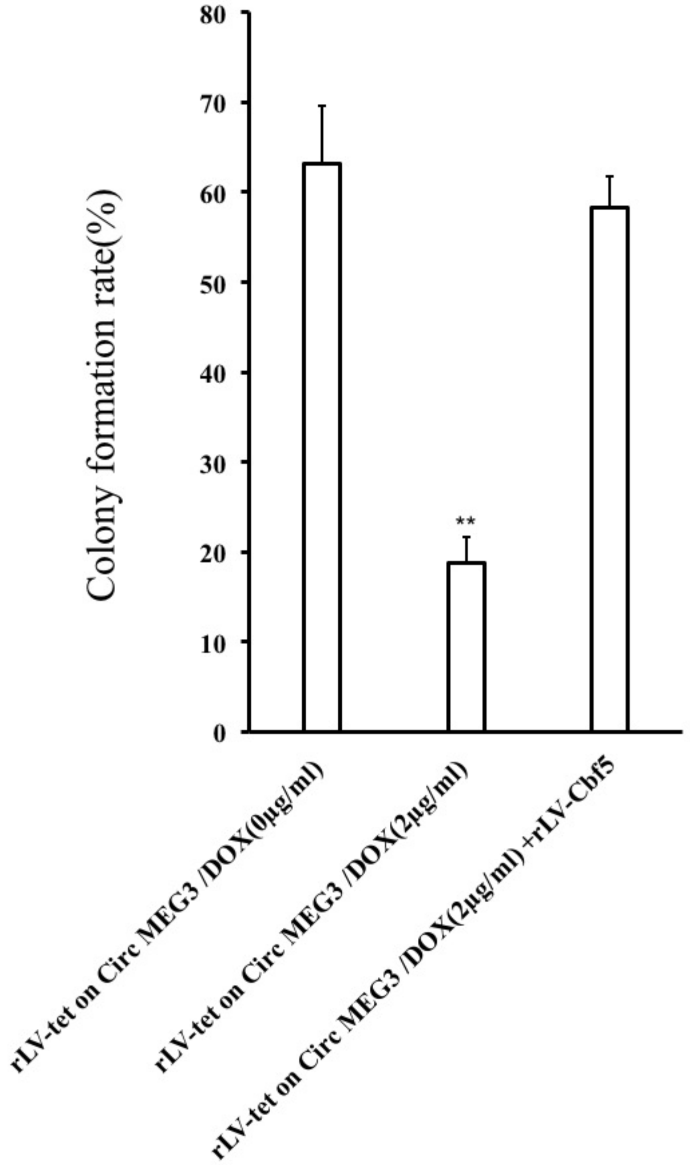

FigureS5. The analysis of of human liver cancer stem cell colony formation ability. **, P <0.01 or *, P <0.05 means statistical difference is significant.

FigureS6

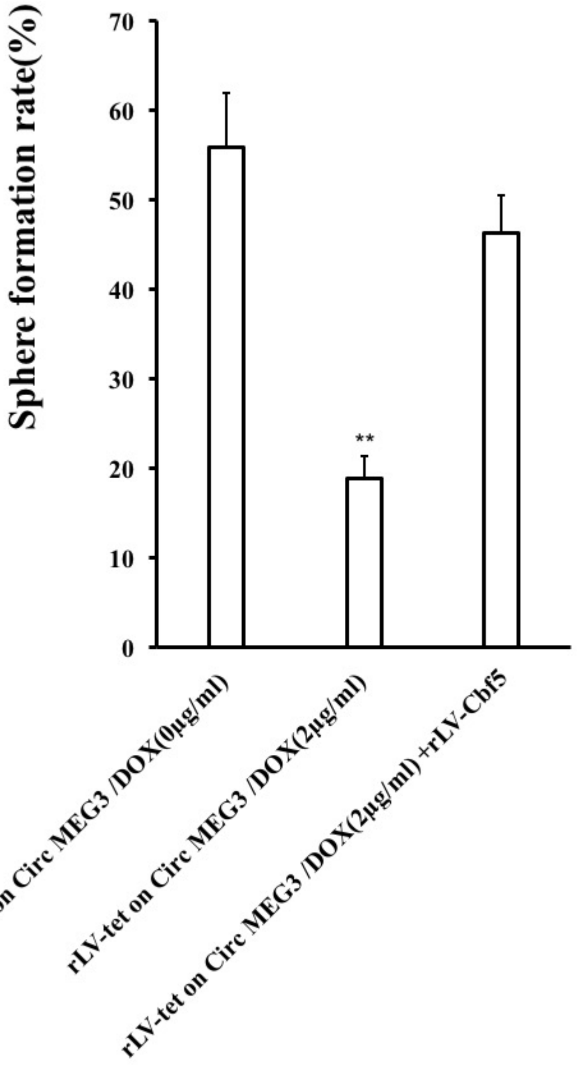

FigureS6 The analysis of human liver cancer stem cell sphere formation rate. **, P <0.01 or *, P <0.05 means statistical difference is significant.
